# Synthesis of Aromatic Rings Embedded in Polycyclic Scaffolds by Triyne Cycloaddition: Competition between Carbonylative and Non-Carbonylative Pathways

**DOI:** 10.3390/molecules24030595

**Published:** 2019-02-07

**Authors:** Laura Salacz, Nicolas Girard, Jean Suffert, Gaëlle Blond

**Affiliations:** Laboratoire d’Innovation Thérapeutique, Université de Strasbourg, CNRS, 67000 Strasbourg, France; laura.salacz@etu.unistra.fr (L.S.); nicolas.girard@unistra.fr (N.G.)

**Keywords:** cyclotrimerization, carbonylative cycloaddition, tropone, benzenoid, polycycles, rhodium catalysis, one-pot reaction

## Abstract

Cycloadditions have emerged as some of the most useful reactions for the formation of polycyclic compounds. The carbonylative cycloaddition of triynes can lead to carbonylative and non-carbonylative competitive pathways, each leading to the formation of an aromatic ring. We report herein the one-pot synthesis of fully- and unsymmetrically-substituted tetracyclic 6,5,7,5-troponic and 6,5,6,5-benzenoid scaffolds using pre-organized triynes showing the competition between these two pathways.

## 1. Introduction

In the past century, the advent of modern organic chemistry has triggered a revolution in the chemical landscape. The vast increase of means of synthesis and analysis has allowed a tremendous diversification of possibilities, including the ability to synthesize complex structures inspired by nature [1]. Due to the polycyclic nature of many of these scaffolds, cycloaddition reactions have been intensively developed. However, these reactions are necessarily limited by Woodward-Hoffmann rules, and are therefore limited to classical ring-sizes [2]. Metal-catalyzed cycloadditions, on the other hand, allow the synthesis of different ring-sizes. Additionally, they can bring together three or more components for the synthesis of exotic structures. Indeed, in recent years, considerable advances have been made in the development of [2+2+2] [3], [2+2+2+1] [4,5], [3+3+1] [6], [5+2+1] [7,8], and various other cycloadditions [9,10,11].

The use of alkynes in cycloaddition reactions allows the formation of unsaturated compounds. They have been extensively used for the synthesis of complex and original structures. Additionally, the intramolecular cyclotrimerization of alkynes has been applied to the synthesis of many useful compounds containing a benzene ring, including helicenes [12]. Nevertheless, examples of the cycloaddition of fully substituted unsymmetrical triynes are scarce in the literature, even though highly substituted and non-symmetrical polycycles have attracted much attention. Moreover, until recently, no example combined the cycloaddition of triynes with carbonylative cycloaddition.

**Scheme 1:**
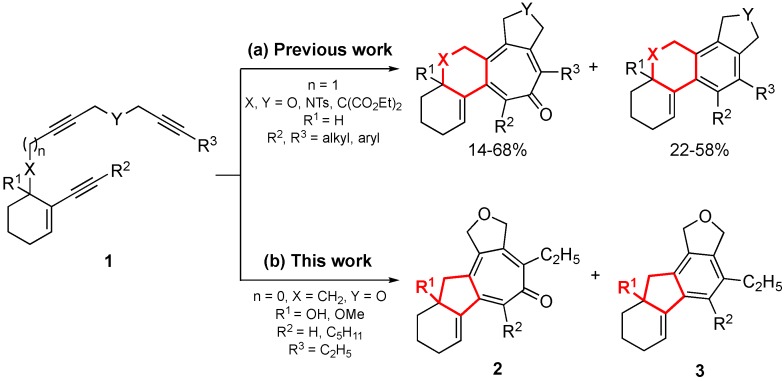
[2+2+2+1] cycloaddition of unsymmetrically fully substituted triynes (**a**) Previous work in our group; (**b**) this work on the competition of carbonylative and non-carbonylative pathways of the reaction of pre-organized triynes containing shorter tethers.

In the course of our work on the synthesis of polycyclic compounds containing 7- and 8-membered rings [13,14,15,16], we recently interested ourselves in the Rh^I^-catalyzed [2+2+2+1] carbonylative cycloaddition of pre-organized triynes yielding 2,4,6-cycloheptatrienones (Scheme 1a) [17]. These compounds, more commonly referred as tropones [18], are non-benzenoid aromatic carbonylated 7-membered rings that have attracted increasing attention due to their interesting electronic properties [19,20,21], their presence in several natural products [22,23], and their 8π system, which gives them importance as synthons [24].

A major challenge with the formation of carbonylated compounds by [2+2+2+1] carbonylative cycloaddition of triynes is the competing byproduct arising from the non-carbonylative pathway. During our seminal work, we hypothesized that favoring the carbonylative pathway could be achieved by using a substrate containing a cyclic core allowing the constriction of the reactant in order to place the reactive moieties at an ideal distance to undergo fast cyclization. With this strategy, we reported the synthesis of fully- and unsymmetrically-functionalized fused 6,6,7,5- and 6,6,6,5-tetracycles obtained through, respectively, the carbonylative and non-carbonylative reaction pathway (Scheme 1a).

Remarkably, during this study, evidence was found that the formation of 6-membered rings from the tethers of the triynes is challenging in this process. In following work, we explored the possibilities offered by tethers leading to the formation of 5-membered cycles (Scheme 1b).

These would allow the formation of novel 6,5,7,5,- and 6,5,6,5-fused tetracycles containing an aromatic tropone or benzenoid moiety. In addition to the generated structural diversity, the envisioned products would comprise an oxygenated quaternary carbon center at a ring junction, allowing post-functionalization as well as the modification of the spatial conformation.

## 2. Results

**Scheme 2:**
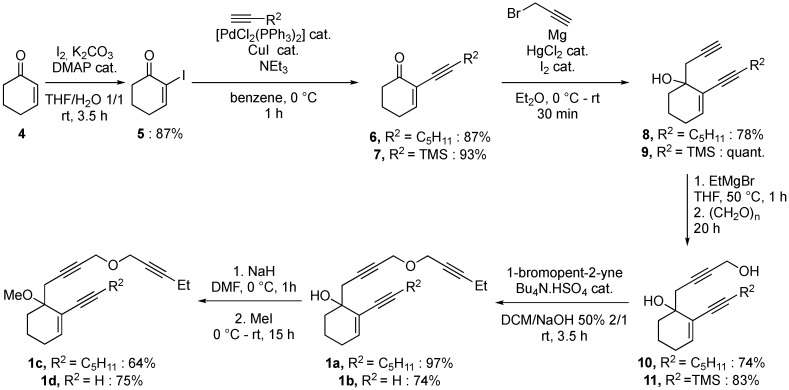
Synthesis of substrates **1a**–**d**.

With the previous results in hand, and having observed that the formation of 6-membered rings is challenging in this process, we chose to investigate the formation of 6,5,7,5-polycyclic tropones and 6,5,6,5-polycylic benzenoids. We therefore synthesized substrates **1a**–**d** (Scheme 2), containing an alkyl tether. Commercial cyclohexenone was first iodinated using the described conditions [25]. The resulting 2-iodocyclohexenone **5** then underwent Sonogashira cross-coupling in standard conditions with 1-heptyne and ethynyltrimethylsilane yielding respectively **6** with 87% yield and **7** with 93% yield. The resulting enynones were then submitted to a Grignard reaction with propargyl bromide in the presence of a catalytic amount of mercuric chloride to avoid polymerization of the reagent, giving tertiary alcohols **8** and **9** in high yields. The terminal alkynes were then deprotonated by ethylmagnesium bromide and reacted with paraformaldehyde to give propargylic alcohols **10** and **11** in 74% and 83% yield, respectively. These underwent Williamson reaction with commercial 1-bromo-2-pentyne in biphasic media, leading to the formation of corresponding substrate **1a** in 97% yield and **1b** through base-mediated desilylation in 74% yield. The free alcohols were methylated selectively by the action of methyl iodide to give pre-organized triynes **1c** and **1d** in good yields.

With these triynes in hand, we investigated the competitive pathways of rhodium-catalyzed [2+2+2+1] and [2+2+2] cycloadditions of 5-atom-tethered-triynes to study the impact of the tether size as well as potential alternative carbon monoxide sources in these processes.

### 2.1. Use of Mo(CO)6 as CO Source for the [2+2+2+1] Carbonylative Cycloaddition

Due to its high toxicity and the necessary setup, and despite its frequent utilization in industry, the use of carbon monoxide is often deemed unpractical by synthetic chemists. Consequently, many alternatives have been developed in recent years. Mo(CO)_6_ is one of the most used metallic CO sources, particularly as a carbonylative cycloaddition catalyst or in palladium-catalyzed carbonylative cycloaddition [26,27,28]. However, to the best of our knowledge, no example combining CO-donation by molybdenum and rhodium-catalyzed carbonylative cycloaddition have been reported. We therefore elected to study the possibility of combining the reactivity of rhodium catalysts in cycloaddition reactions with the CO-donating ability of molybdenum complex Mo(CO)_6_.

In a first experiment, substrate **1a** was used with 10 equivalents of molybdenum hexacarbonyl and 5 mol% of rhodium catalyst (Table 1, entry 1). In this case, tropone **2a** and benzenoid **3a** were obtained in a 1/3 ratio, respectively, and the formation of a third compound, **12**, by [2+2+2] cyclotrimerization and dehydration was also observed. Interestingly, the dehydrated troponic analog of **2a** was not detected, indicating that dehydration and carbon monoxide insertion are mutually exclusive in these conditions. This experiment seems to indicate that no ligand transfer from the molybdenum to the rhodium takes place and that all the CO ligands of the rhodium catalyst undergo migratory insertion. This observation could be explained in the presence of a strongly coordinating solvent, however, in this case, no satisfactory explanation could be found.

A second experiment without molybdenum was done and reached full conversion overnight (entry 2). However, we observed similar results as those observed in entry 1, confirming our first hypothesis that all the CO insertion results from the rhodium catalyst.

Then using high loading catalyst (25 mol% of [Rh(CO)_2_Cl]_2_) at room temperature, full conversion was achieved in 10 min and in this case only one carbon monoxide ligand per molecule of catalyst was inserted in the products (entry 3).

Another experiment using only Mo(CO)_6_ lead to the sole formation of dehydrated compound **12**, indicating that the molybdenum complex does not enable CO migratory insertion (entry 4). However, ligand exchange between CO and alkyne moieties seems to occur, as this step is necessary for the formation of compound **12**. Moreover, comparison of entries 1 and 4 indicates that rhodium has a higher affinity for the multiple bonds, as the formation of **12** is not a competing process whether Mo(CO)_6_ is present in the media or not (entries 1, 2 and 4). In order to verify that CO transfer between molybdenum and rhodium does not occur, we conducted two additional experiments in toluene at higher temperature under micro-wave irradiation (entries 5 and 6). In both cases, the amount of tropone **2a** obtained was consistent with very low or no participation of the molybdenum complex, and only the yield of dehydrated compound **12** increased, as can be expected at a higher temperature.

In other words, our experiments showed that, in these conditions, Mo(CO)_6_ is not efficient as a CO source in rhodium-catalyzed [2+2+2+1] carbonylative cycloaddition of triynes. Additionally, an equilibrium of approximately 1/3 in favor of the non-carbonylative pathway is observed in the presence of the rhodium catalyst, catalytic loading notwithstanding. Moreover, we observed the emergence of a new competitive [2+2+2]/elimination pathway. However, a higher content in metal complex in the media and a higher temperature favor the dehydration process, indicating a Lewis-acid mediated reaction.

### 2.2. Use of Carbon Monoxide Gas as CO Source for the [2+2+2+1] Carbonylative Cycloaddition

In order to avoid the formation of a third compound by dehydration and increase molecular diversity, methoxy-protected substrates **1c** and **1d** were used to study the rhodium-catalyzed formation of 6,5,7,5- and 6,5,6,5-fused tetracycles.

A first experiment using 5 mol% of rhodium catalyst was conducted on substrates **1c** and **1d** (Table 2, entries 1 and 2). Identically to substrate **1a**, a 1/3 ratio of carbonylated compounds **2c**,**d** and [2+2+2] cycloaddition compounds **3c**,**d** is observed. Despite the protection of the alcohol functionality, a low amount of demethoxylation products **12**–**13** is observed. Because Mo(CO)_6_ is not efficient as a CO source, the use of carbon monoxide was considered. Under 2 atm of CO, the proportion of carbonylative pathway increases, as does the demethoxylation (entries 3 and 4). It is probable that a higher pressure of CO reduces the electron density on the rhodium due to retrodonation of the rhodium to the CO ligand, increasing the Lewis-acid character and thus enabling the elimination. This supposition is supported by entry 5, where 10 atm of CO lead to the formation of **12** as the major product. This entry also supports previous studies indicating that a higher pressure hinders the rhodium-catalyzed carbonylative cycloaddition processes [29]. In order to enrich the media in carbon monoxide without hampering the carbonylative pathways, we bubbled the media with carbon monoxide for 15 min before applying pressure in a closed vessel (entry 6). Interestingly, full conversion was not achieved overnight and an additional 8 h were necessary to reach completion (entry 7). In these conditions, the products **2c**, **3c** and **12** are obtained in equimolar ratio. When the carbon monoxide is bubbled in a solution containing only the catalyst prior to the addition of the substrate, the carbon monoxide insertion increases, and the demethoxylative pathway becomes the minority (entry 8). The conversion, however, is lowered, which seems to indicate a saturation of the catalyst by the CO. The same tendency can be observed when the CO is bubbled in the media containing only the substrate prior to the addition of the catalyst (entry 9).

With substrates **1c**,**d** we were able to synthesize five new tetracyclic compounds containing aromatic rings, as well as to observe the emergence of a new competitive [2+2+2]/elimination pathway. The latter is directly dependent on the employed CO-pressure, possibly due to a stronger Lewis-acidity of the rhodium catalyst. Moreover, the proportion of this process, in comparison to that of the simple [2+2+2] cycloaddition, increases when the media is saturated in carbon monoxide by bubbling.

### 2.3. Mechanistic Proposals

**Scheme 3:**
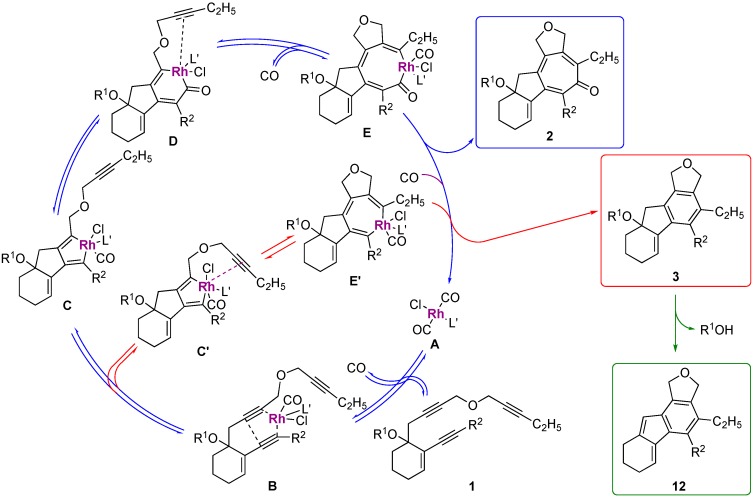
Proposed mechanisms for the formation of compounds **2**, **3**, and **12**.

Scheme 3 summarizes the proposed mechanism for carbonylative cycloaddition, cyclotrimerization and cyclotrimerization/elimination processes based on previous studies [17,30]. It is unsure if the dimeric pre-catalyst first undergoes ligand exchange with CO to form catalytic species [RhCl(CO)_3_] or if the coordination bond with the bridging chloride is preserved. L’ in **A** can therefore be another CO ligand or the second half of the dimeric catalyst. The two alkyne moieties of substrate **1**, ideally placed by the cyclic core, then coordinate to the metallic center, displacing the CO ligands and forming intermediate **B**. This 18-electron complex can undergo a [2+2+M] cyclometallation, yielding either a 16-electron complex **C** or an 18-electron complex **C′**, which is stabilized by the chelation of the third alkyne moiety. Migratory insertion of CO can take place in the Rh-Csp^2^ bond of intermediate **C** to access intermediate **D**, where the electron-deficient complex is stabilized by the chelation of the third alkyne. This triple bond undergoes migratory insertion, forming 8-membered rhodacycle **E**, which, after reductive elimination, releases troponic compound **2** and catalytic species **A**. Complex C′, being already chelated by the third alkyne moiety, can undergo direct migratory insertion of the multiple bond, yielding 7-membered rhodacycle **E′**. DFT calculations on the [2+2+2+1] carbonylative cycloaddition of enediynes have shown that late stage CO insertion is highly disfavored, thus **E′** undergoes direct reductive elimination to release benzenoid compound **3** in the media [31,32].

Compound **3** can then undergo a rhodium-mediated elimination, which is favored when the CO pressure, and consequently the catalyst’s Lewis-acidic properties, are increased. This late-stage elimination pathway is favored by the complete conjugation of byproduct **12**. With the aromatic properties of tropones residing in their mesomeric forms, this conjugation is only possible in resonance extremes, and therefore the elimination does not occur. Conceptually, it is possible to imagine that this elimination process takes place prior to the cycloaddition reaction. However, in that case, the dehydrated analog of **2** should be observed. Indeed, both carbonylative and non-carbonylative pathways go through intermediate **B**, and if the formation of the benzene ring from there is possible when the substrate possesses an additional unsaturation, the carbonylative reaction should equally take place.

This mechanism allows the rationalization of the observed reaction rates depending on the length of the tethers. The formation of **C** and **C′** are possible overnight at room temperature with tethers leading to the formation of 5-membered rings, yet the reaction is sluggish when this tether is homologated [17].

## 3. Materials and Methods

Procedures for the synthesis of the substrates and the products and full characterization of final products can be found in the Supplementary Information.

## 4. Conclusions

To conclude, we have successfully investigated the rhodium-catalyzed cycloaddition reactions of pre-organized triynes for the synthesis of various and original tetracyclic scaffolds. These compounds, containing an aromatic motif that can be either a tropone or a benzene ring and are fully unsymmetrical, have been synthesized with very high combined yields of 80–100%. We have been able to synthesize three examples of 6,5,7,5-structures and four examples of 6,5,6,5-structures. 

Additionally, we have explored the use of Mo(CO)_6_ as an alternative CO donor. Unfortunately, these experiments have shown that Mo(CO)_6_ is not a suitable CO source in these processes, due to a lack of ligand exchange. On other hand, a new competitive dehydration/cycloisomerization pathway has been observed and rationalized in terms of Lewis-acidity of the rhodium catalyst. Moreover, the molybdenum species shows a definite selectivity for this pathway, whereas, when combined with a rhodium catalyst, dehydration becomes minority, due to competition between the metals.

## Figures and Tables

**Table 1:**
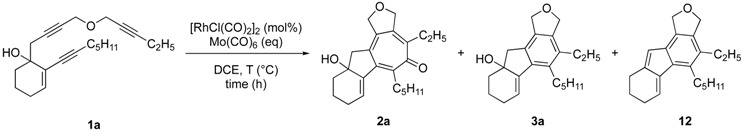
Use of Mo(CO)_6_ as CO source for the [2+2+2+1] carbonylative cycloaddition and competing pathways of triyne **1a**.

Entry	[Rh] (mol%)	Mo(CO)_6_ (eq)	Temperature (°C)	Time (h)	Conv. (%)	2a ^a^	3a ^a^	12 ^a^
1 ^b^	5	10	rt	18	100	24 (19)	71 (41)	5 (5)
2	5	-	rt	18	100	25	75	0
3	25	-	rt	0.17	100	(27)	(43)	(30)
4 ^b^	-	10	rt	18	90	0	0	90
5 ^c^	5	10	50	0.75	100	25	62	13
6 ^c^	5	10	100	0.5	89	23	54	17

^a 1^H NMR ratio, isolated yield in brackets; ^b^ reaction conducted in DCE; ^c^ reaction conducted in toluene under MW irradiation.

**Table 2:**
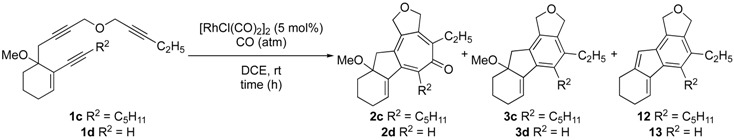
Use of carbon monoxide as CO source for the [2+2+2+1] carbonylative cycloaddition and competing pathways of triyne **1c**,**d**.

Entry	R^2^	P_CO_ (atm)	Time (h)	Conversion (%)	2 ^a^	3 ^a^	12, 13 ^a^
1	**1c**	-	18	100	**2c**, 22	**3c**, 73	**12**, 0
2	**1d**	-	18	100	**2d**, 25	**3d**, 75	**13**, <5
3	**1c**	2	18	100	**2c**, 33	**3c**, 46	**12**, 21
4	**1d**	2	18	100	**2d**, 29 (29)	**3d**, 43 (43)	**13**, 17
5	**1c**	10	18	100	**2c**, 25	**3c**, 31	**12**, 44
6 ^b^	**1c**	2	18	73	**2c**, 26	**3c**, 25	**12**, 22
7 ^b^	**1c**	2	26	100	**2c**, 34 (17)	**3c**, 32 (24)	**12**, 33 (20)
8 ^c^	**1c**	2	26	90	**2c**, 33	**3c**, 33	**12**, 23
9 ^d^	**1c**	2	26	91	**2c**, 36	**3c**, 32	**12**, 23

^a 1^H NMR ratio, isolated yield in brackets; ^b^ media bubbled with CO for 15 min; ^c^ solution of catalyst bubbled with CO for 15 min, then substrate was added; ^d^ solution of substrate bubble with CO for 15 min, then catalyst was added.

## Supplementary Materials

Supplementary Materials are available online.
