# Supplementary material for: Synthesis of Aromatic Rings Embedded in Polycyclic Scaffolds by Triyne Cycloaddition: Competition between Carbonylative and Non-Carbonylative Pathways
Source: Molecules. 2019 Feb 7;24(3):595. doi: 10.3390/molecules24030595 (PMC6384885; doi:10.3390/molecules24030595)
Supplement: Supplementary file 1 [file molecules-24-00595-s001.pdf]

Supplementary information

Table of contents

General information 2

General procedures 2

Synthesis of substrates **1** and products **2**, **3** and **12** 4

Spectras 12

1a 12

1b 13

1c 14

1d 15

2a 16

2c 17

2d 18

3a 19

3c 20

3d 21

6 22

8 23

9 24

10 25

11 26

12 27

References 28

General information

All reagents, chemicals and dry solvents were purchased from commercial sources and used without purification. When mentioned that the reaction was conducted in dry media, glassware dried for several hours at 110 °C in an oven was used. Triethylamine (Et_3N) and diisopropylamine (DIPA) were distilled on KOH in an S-tube prior to each experiment in which they were involved. Reactions were monitored by TLC (Thin Layer silica gel Chromatography) using Merck silica gel 60 F254 on aluminum sheets. TLC plates were visualized under UV light and revealed with acidic *p*-anisaldehyde stain or $KMnO_4$ stain. Crude products were purified by flash column chromatography on Merck silica gel Si 60 (40-63 µm). NMR spectra were recorded in $CDCl_3$ on a Bruker Avance III BBFO+ probe spectrometer 400 MHz for ^{1}H analyses and 100 MHz for ^{13}C analyses. Proton chemical shifts are reported in ppm (δ), relatively to residual $CHCl_3$ (δ 7.26 ppm). Multiplicities are reported as follows: singlet (s), doublet (d), triplet (t), quartet (q), quintet (quint), broad singlet (bs), broad doublet (bd) combinations or multiplet (m). Coupling constants values *J* are given in Hz. Carbon chemical shifts are reported in ppm (δ), relatively to the internal standard $CDCl_3$ (δ 77.23 ppm). ^{1}H and ^{13}C NMR signals were assigned mostly on the basis of 2D-NMR (COSY, HSQC, HMBC) experiments. High Resolution Mass Spectral analyses (HRMS) were performed using an Agilent 1200 RRLC HPLC chain and an Agilent 6520 Accurate mass QToF. Infrared spectra (IR) were recorded on a FT IR Thermo Nicollet ATR 380, Diamant Spectrometer.

General procedures

General procedure I: Williamson reaction

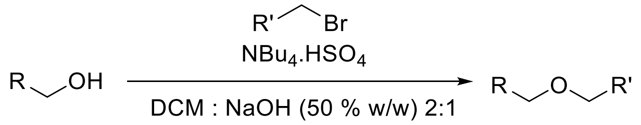

10, 11 **1**

The alcohol (1 eq.) was dissolved in DCM (0.14 M), then tetrabutylammonium hydrogen sulfate (0.2 eq.) was added. Alkyl bromide (3.1 eq.) and a solution of NaOH 50% w/w in water (0.28 M) were added. The mixture was heated at 40 °C under vigorous stirring and monitored by TLC. Alkyl bromide (3 eq.) was added when necessary. After completion, the mixture was diluted with water, extracted 3 times with DCM and the organics were dried over $MgSO_4$ and concentrated *in vacuo*. The crude was purified by silica flash chromatography (pentane:AcOEt 9:1).

General procedure II: synthesis of enynones

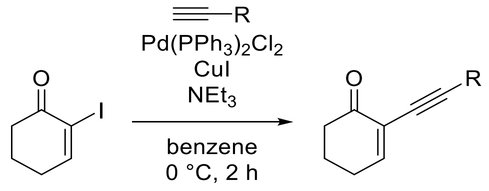

5 **6, 7**

2-iodocyclohexenone **5** (1 eq.), copper iodide (4 mol%) and $PdCl_2(PPh_3)_2$ (2 mol%) chloride were suspended in benzene (0.22 M) under argon in a dry flask. Freshly distilled Et_3N (4.6 eq.) was added and the mixture was frozen in liquid nitrogen. Oxygen was removed by putting the flask under vacuum followed by purging with argon. The mixture was then warmed to room temperature, then frozen again. The freeze-purge-defreeze cycle was repeated three times. Then, the terminal alkyne (2 eq.) was added and the mixture was stirred for 2 h at 0 °C. After completion, the reaction was poured over Et_2O, washed with $NaHCO_3$, and the organics were extracted twice with Et_2O. The organics were dried over $MgSO_4$, filtered and concentrated *in vacuo*. The crude mixture was purified using silica flash chromatography (pentane:AcOEt 9:1).

General procedure III: Grignard reaction on enynones

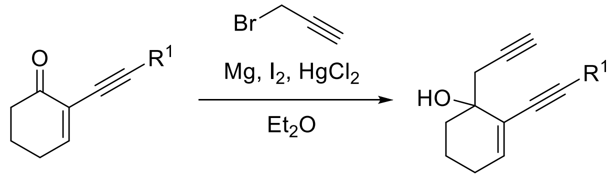

6, 7 **8, 9**

Magnesium turnings (1.8 eq.) were added in a dry flask under argon then dry Et_2O was added (2 M with respect to the substrate). Then, $HgCl_2$ (5 mol%) and iodine (5 mol%) were added. The mixture become red. A few drops of propargyl bromide 80% in toluene were added. A precipitate appears. The mixture was allowed to stir 5 minutes at room temperature then cooled to 0 °C. The same quantity of Et_2O as previously was added in the flask. A mixture of propargyl bromide (1.5 eq.) and enynone **6** or **7** (1 eq.) were dissolved in the same amount of dry Et_2O and this solution was added to the cooled mixture. 4 times the starting amount of Et_2O was then added to the mixture, and it was warmed to room temperature and stirred vigorously for 30 minutes. Remaining magnesium turnings were then filtered on cotton and the mixture was poured over saturated $NaHCO_3$, extracted twice with Et_2O, dried over $MgSO_4$ and concentrated *in vacuo*. The crude was purified by silica flash chromatography (pentane:AcOEt 9:1)

General procedure IV: synthesis of propargyl alcohols from terminal alkynes

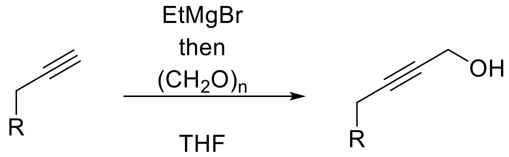

8,9 **10,11**

In a dry flask under argon fitted with a condenser, terminal alkyne (1 eq.) was dissolved in dry THF (0.1 M). EtMgBr 40% in 2-methyltetrahydrofuran (5 eq.) was added and the mixture was heated to 50 °C for 1 h. Then, paraformaldehyde (1.33 eq.) was added and the mixture was stirred at 50 °C for 23 h. The mixture was then allowed to cool to room temperature and quenched with sat. $NaHCO_3$ and extracted 3 times with Et_2O. The organics were dried over $MgSO_4$ and the crude was purified using silica flash chromatography (pentane:AcOEt 9:1).

Synthesis of substrates 1 and products 2, 3 and 12

Synthesis of substrates 1a-d

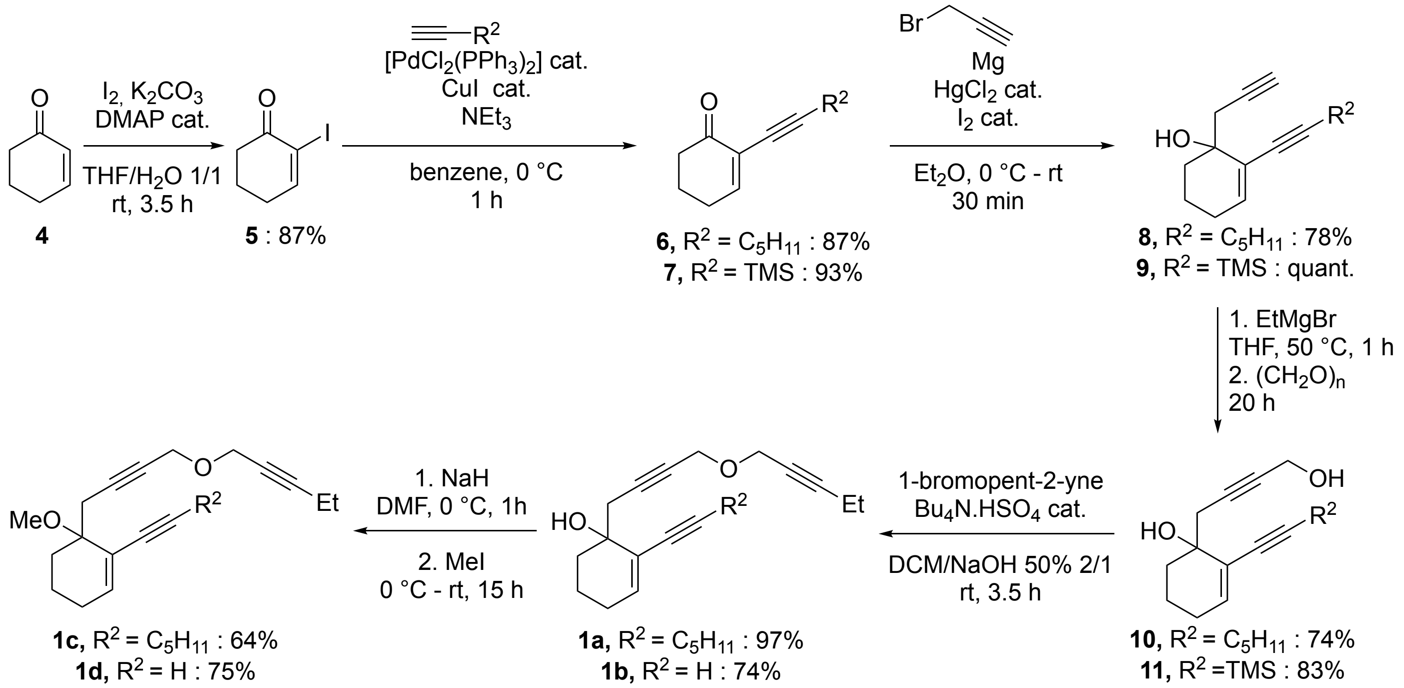

1a. 2-(hept-1-yn-1-yl)-1-(4-(pent-2-yn-1-yloxy)but-2-yn-1-yl)cyclohex-2-en-1-ol

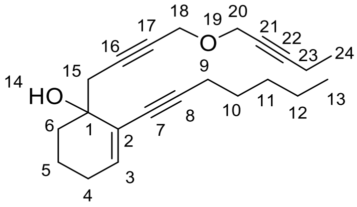

Chemical Formula: $C_{22}H_{30}O_2$
Molecular Weight: 326,48

Compound **1a** was synthesised following general procedure I using **10** (572.2 mg, 2.198 mmol, 1 eq.), NaOH 50% in water (5 mL), 1-bromopent-2-yne (0.25 mL, 2.417 mmol, 1.1 eq.), $Bu_4N.HSO_4$ (76.9 mg, 0.220 mmol, 0.1 eq.) and DCM (12 mL). Reaction was over after 3.5 hours. Work-up and purification afforded **1a** as a clear oil in 97% yield (692.5 mg, 2.121 mmol).

Rf = 0.66 (pentane:AcOEt 7:3) **^{1}H NMR (400 MHz, CDCl$_3$):** δ 6.15 (t, *J* = 4.0 Hz, 1H, H3), 4.24 (s, 2H, H18), 4.21 (s, 2H, H20), 2.81, 2.58 (ABq, *J* = 16.7 Hz, 2H, H15), 2.32 (t, *J* = 7.2 Hz, 2H, H9), 2.23 (q, *J* = 7.5 Hz, 2H, H23), 2 .12 (m, 2H, H4), 2.07 (bs, 1H, H14), 1.95 (m, 1H, H6), 1.86 (m, 1H, H6'), 1.73 (m, 1H, H5), 1.65 (m, 1H, H5'), 1.55 (m, 2H, H10), 1.40-1.30 (m, 4H, H11, H12), 1.14 (t, *J* = 7.5 Hz, 3H, H24), 0.90 (t, *J* = 6.8 Hz, 3H, H13) **^{13}C NMR (100 MHz, CDCl$_3$):** δ 136.8 (C3), 126.0 (C2), 91.6 (C8), 88.8 (C22), 82.3 (C16), 78.2 (C17), 77.8 (C7), 74.7 (C21), 70.3 (C1), 57.0 (2C, C18, C20), 34.0 (C6), 31.7 (C15), 31.2 (C11), 28.5 (C10), 25.9 (C4), 22.2 (C12), 19.3 (C9), 18.7 (C5), 14.0 (C13), 13.8 (C24), 12.5 (C23) **HRMS :** (ESI)+ : Calculated for $[C_{22}H_{31}O_2]^+$: 327.2324, found : 327.23161 (Diff. : 0.74 ppm)

1b. 2-ethynyl-1-(4-(pent-2-yn-1-yloxy)but-2-yn-1-yl)cyclohex-2-en-1-ol

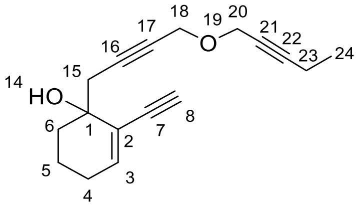

Chemical Formula: $C_{17}H_{20}O_2$
Molecular Weight: 256,35

Compound **1b** was synthesised following general procedure I using **11** (623.5 mg, 2.376 mmol, 1 eq.), NaOH 50% in water (5.2 mL), 1-bromopent-2-yne (0.26 mL, 2.495 mmol, 1.05 eq.), $Bu_4N.HSO_4$ (83.2 mg, 0.238 mmol, 0.1 eq.) and

DCM (12.4 mL). Reaction was over after 2.5 hours. Work-up and purification afforded **1b** as a clear oil in 74% yield (451.8 mg, 1.763 mmol).

Rf = 0.68 (pentane:AcOEt 7:3)**^{1}H NMR (400 MHz, $CDCl_3$):** δ 6.36 (t, *J* = 4.0 Hz, 1H, H3), 4.23 (s, 2H, H18), 4.21 (s, 2H, H20), 2.94 (s, 1H, H8), 2.83, 2.62 (ABq, *J* = 16.9 Hz, 2H, H15), 2.23 (q, *J* = 7.7 Hz, 2H, H23), 2 .16-2.05 (m, 3H, H4, H14), 1.96 (m, 1H, H6), 1.87 (m, 1H, H6'), 1.74 (m, 1H, H5), 1.67 (m, 1H, H5'), 1.14 (t, *J* = 7.7 Hz, 3H, H24), **^{13}C NMR (100 MHz, $CDCl_3$):** δ 140.4 (C3), 124.9 (C2), 88.8 (C22), 82.3 (C16), 81.3 (C7), 78.5 (C17), 78.3 (C8), 74.6 (C21), 70.0 (C1), 57.0 (C18), 56.9 (C20), 34.2 (C6), 31.6 (C15), 26.0 (C4), 18.5 (C5), 13.5 (C23), 12.5 (C24). **HRMS :** (ESI)+ : Calculated for $[C_{17}H_{20}O_2Na]^+$: 279.1361, found : 279.1343 (Diff. : 4.41 ppm) **IR:** ν 3444, 3281, 2937, 2358, 1439, 1348, 1134, 1069, 987, 975, 734

1c. 1-(hept-1-yn-1-yl)-6-methoxy-6-(4-(pent-2-yn-1-yloxy)but-2-yn-1-yl)cyclohex-1-ene

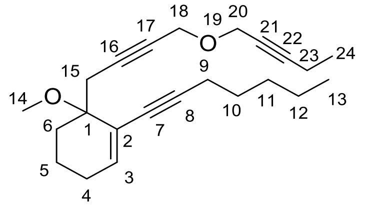

To a suspension of NaH 60% in mineral oil (182 mg, 4.55 mmol, 1.5 eq.) in dry DMF (15 mL) under argon at 0 °C was added a solution of **1a** (990.3 mg, 3.033 mmol, 1 eq.) in dry DMF (15 mL) dropwise. The mixture was stirred for 1 h then methyl iodide (0.57 mL, 9.1 mmol, 3 eq.) was added dropwise. The mixture was allowed to reach room temperature and stirred overnight. It was then quenched with water, extracted 4 times with AcOEt, dried over $MgSO_4$ and concentrated *in vacuo.* Purification by silica flash chromatography (pentane:AcOEt 95:5) afforded **1c** as a yellow oil in 64% yield (665 mg, 1.953 mmol).

Rf = 0.57 (pentane:AcOEt 9:1) **^{1}H NMR (400 MHz, $CDCl_3$):** δ 6.28 (t, *J* = 4.0 Hz, 1H, H3), 4.23 (s, 2H, H18), 4.21 (s, 2H, H20), 3.29 (s, 3H, H14), 2.80, 2.62 (ABq, *J* = 16.8 Hz, 2H, H15), 2.30 (t, *J* = 7.0 Hz, 2H, H9), 2.22 (q, *J* = 7.7 Hz, 2H, H23), 2 .09 (m, 2H, H4), 1.96 (m, 1H, H6), 1.86 (m, 1H, H6'), 1.75 (m, 1H, H5), 1.67 (m, 1H, H5'), 1.53 (m, 2H, H10), 1.40-1.29 (m, 4H, H11, H12), 1.14 (t, *J* = 7.7 Hz, 3H, H24), 0.89 (t, *J* = 7.0 Hz, 3H, H13) **^{13}C NMR (100 MHz, $CDCl_3$):** δ 139.3 (C3), 124.3 (C2), 90.5 (C8), 88.6 (C22), 83.8 (C16), 78.9 (C17), 77.5 (C7), 75.2 (C1), 74.8 (C21), 57.1 (C18), 56.9 (C20), 51.2 (C14), 31.1 (C11), 30.4 (C6), 29.2 (C15), 28.6 (C10), 25.9 (C4), 22.2 (C12), 19.4 (C9), 19.1 (C5), 14.0 (C13), 13.8 (C24), 12.5 (C23). **HRMS :** (ESI)+ : Calculated for $[C_{23}H_{32}O_2Na]^+$: 363.4968, found : 363.2284 (Diff. : 3.19 ppm) **IR:** ν 2934, 2858, 1457, 1344, 1348, 1319, 1135, 1070, 893, 729.

1d. 1-ethynyl-6-methoxy-6-(4-(pent-2-yn-1-yloxy)but-2-yn-1-yl)cyclohex-1-ene

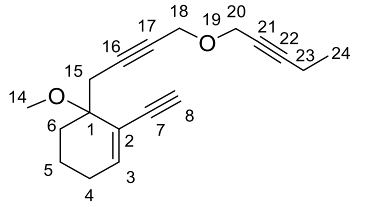

To a suspension of NaH 60% in mineral oil (136.2 mg, 3.406 mmol, 1.5 eq.) in dry DMF (10 mL) under argon at 0 °C was added a solution of **1b** (582 mg, 2.27 mmol, 1 eq.) in dry DMF (10 mL) dropwise. The mixture was stirred for 1 h then methyl iodide (0.43 mL, 6.811 mmol, 3 eq.) was added dropwise. The mixture was allowed to reach room temperature and stirred overnight. It was then quenched with water, extracted 4 times with AcOEt, dried over $MgSO_4$ and concentrated *in vacuo.* Purification by silica flash chromatography (pentane:AcOEt 98:2 to 85:15) afforded **1d** as a yellow oil in 75% yield (458.9 mg, 1.697 mmol).

Rf = 0.69 (pentane:AcOEt 95:5)**^{1}H NMR (400 MHz, $CDCl_3$):** δ 6.51 (t, *J* = 4.0 Hz, 1H, H3), 4.23 (s, 2H, H18), 4.21 (s, 2H, H20), 3.31 (s, 3H, H14), 2.90 (s, 1H, H8), 2.79, 2.67 (ABq, *J* = 16.8 Hz, 2H, H15), 2.23 (q, *J* = 7.5 Hz, 2H, H23), 2 .17-2.08 (m, 2H, H4), 1.96 (m, 1H, H6), 1.89 (m, 1H, H6'), 1.77 (m, 1H, H5), 1.71 (m, 1H, H5'), 1.14 (t, *J* = 7.5 Hz, 3H, H24) **^{13}C NMR (100 MHz, $CDCl_3$):** δ 142.8 (C3), 123.2 (C2), 88.7 (C22), 83.4(C16), 82.2 (C7), 77.7 (C17), 77.2 (C8), 75.0 (C1), 74.7 (C21), 57.0 (C18), 56.9 (C20), 51.2 (C14), 30.2 (C6), 29.2 (C15), 26.0 (C4), 19.0 (C5), 13.8 (C23), 12.5 (C24) **HRMS :**

(ESI)+ : Calculated for $[C_{18}H_{22}O_2Na]^+$: 293.1517, found : 293.1507 (Diff. : 1.65 ppm) **IR:** ν 2937, 1453, 1348, 1134, 1067, 977, 890, 649

2a. 4-ethyl-10a-hydroxy-6-pentyl-1,8,9,10,10a,11-hexahydrobenzo[1,2]azuleno[4,5-c]furan-5(3H)-one

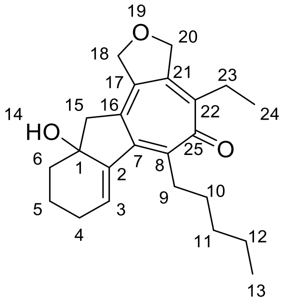

Chemical Formula: $C_{23}H_{30}O_3$
Molecular Weight: 354,49

To a solution of **1a** (50 mg, 0.153 mmol, 1 eq.) in dry DCE (3 mL) under argon, $[Rh(CO)_2Cl]_2$ (14.9 mg, 0.038 mmol, 0.25 eq.) was added. The mixture was stirred at room temperature for 10 min then concentrated *in vacuo* and purified by silica flash chromatography (pentane:AcOEt 85:15 to 1:1) to afford **2a** as a yellow foam in 27% yield (14.7 mg, 0.041 mmol), **3a** as a light yellow oil in 43% yield (21.5 mg, 0.066 mmol) and **12** as a bright yellow oil in 30% yield (14.2 mg, 0.046 mmol).

Rf = 0.34 (pentane:AcOEt 85:15) **^{1}H NMR (400 MHz, $CDCl_3$):** δ 6.26 (bt, *J* = 4.0 Hz, 1H, H3), 5.03, 4.98 (ABq, *J* = 14.0 Hz, 2H, H20), 4.92, 4.79 (ABq, *J* = 13.6 Hz, 2H, H18), 3.22 (ddd, *J* = 11.4, 5.4, 5.4 Hz, 1H, H9), 2.73, 2.64 (ABq *J* = 17.0 Hz, 2H, H15), 2.59 (m, 2H, H9', H23), 2.46 (m, 2H, H23', H4), 2.23 (m, 2H, H4', H6), 2.00 (bs, 1H, H14), 1.82 (m, 2H, H5), 1.75 (m, 1H, H10), 1.56 (m, 1H, H6'), 1.37 (m, 4H, H11, H12), 1.25 (m, H10'), 1.11 (t, *J* = 7.2 Hz, 3H, H24), 0.91 (t, *J* = 6.4 Hz, 3H, H13) **^{13}C NMR (100 MHz, $CDCl_3$):** δ 184.0 (C25), 148.1 (C8), 144.4 (C7), 143.6 (C2), 143.3 (C21), 142.9 (C22), 137.5 (C17), 137.0 (C16), 131.3 (C3), 74.0 (C18), 73.9 (C20), 73.7 (C1), 46.8 (C15), 34.3 (C6), 33.0 (C9), 31.6 (C11), 28.4 (C10), 25.1 (C4), 25.3 (C23), 22.0 (C12), 17.1 (C5), 13.6 (C13), 12.0 (C24) **HRMS :** (ESI)+ : Calculated for $[C_{23}H_{30}O_3]^+$: 355.2273, found : 355.22700 (Diff. : -0.62 ppm) **IR:** ν 3410, 2928, 2868, 1359, 2341, 1541, 1456, 1380, 1203, 1073, 995, 941, 731

2c. 4-ethyl-10a-methoxy-6-pentyl-1,8,9,10,10a,11-hexahydrobenzo[1,2]azuleno[4,5-c]furan-5(3H)-one

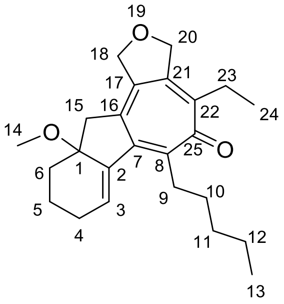

Chemical Formula: $C_{24}H_{32}O_3$
Molecular Weight: 368,52

1c (51.1 mg, 0.150 mmol, 1 eq.) was weighted in a dry vial, then degassed DCE (2.9 mL) was added. Then, $[Rh(CO)_2Cl]_2$ (2.9 mg, 0.008 mmol, 5 mol%) was added and the mixture was bubbled for 15 minutes with CO. The vial was then placed in an oven-dried stainless-steel autoclave under argon, which was purged 3 times with CO then put under a pressure of 2 atm. It was stirred at room temperature for 26 h, then concentrated *in vacuo* and the crude was purified by silica flash chromatography (heptane:AcOEt 9:1 to 6:4). This afforded compound **2c** in 17% yield as a yellow foam (9.4 mg, 0.025 mmol), **3c** in 24% yield as a yellow oil (12.4 mg, 0.036 mmol) and **12** in 20% yield (9.4 mg, 0.030 mmol).

Rf = 0.16 (pentane:AcOEt 85:15) **^{1}H NMR (400 MHz, $CDCl_3$):** δ 6.36 (bt, *J* = 3.2 Hz, 1H, H3), 5.03, 4.99 (ABq, *J* = 14.0 Hz, 2H, H20), 4.92, 4.80 (ABq, *J* = 13.6 Hz, 2H, H18), 3.35 (ddd, *J* = 11.5, 11.5, 5.1 Hz, 1H, H9), 3.12 (s, 3H, H14), 2.82, 2.50 (ABq, *J* = 16.6 Hz, 2H, H15), 2.67 (ddd, *J* = 11.5, 11.5, 4.4 Hz, 1H, H9'), 2.58, (m, 2H, H4, H23), 2.45 (m, 1H, H23), 2.46 (m, 2H, H23', H4), 2.36 (m, 1H, H6), 2.23 (m, 1H, H4'), 1.82-1.72 (m, 3H, H5, H10), 1.38 (m, 5H, H6', H11, H12), 1.25 (m, 1H, H10'), 1.11 (t, *J* = 7.2 Hz, 3H, H24), 0.90 (t, *J* = 6.8 Hz, 3H, H13) **^{13}C NMR (100 MHz, $CDCl_3$):** δ 184.7 (C25), 147.5 (C8), 146.2 (C2), 144.0 (C17), 143.3 (C21), 142.6 (C7), 138.0 (C16), 136.8 (C22), 133.5 (C3), 78.8 (C1), 74.5

(C18), 74.3 (C20), 51.4 (C14), 43.2 (C15), 33.7 (C9), 32.2 (C11), 31.4 (C6), 28.8 (C10), 26.6 (C4), 25.9 (C23), 22.5 (C12), 17.8 (C5), 14.2 (C13), 12.5 (C24) **HRMS :** (ESI)+ : Calculated for $[C_{24}H_{33}O_3]^+$: 369.2430, found : 369.24243 (Diff. : -0.02 ppm) **IR:** ν 2929, 2869, 1556, 1455, 1381, 1072, 908, 728, 646

2d. 4-ethyl-10a-methoxy-1,8,9,2929, 2869, 1555, 1454, 1380, 1205, 1072, 908, 72710,10a,11-hexahydrobenzo[1,2]azuleno[4,5-c]furan-5(3H)-one

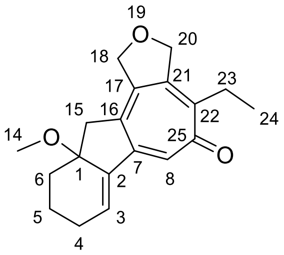

Chemical Formula: $C_{19}H_{22}O_3$
Molecular Weight: 298,38

1d (20 mg, 0.073 mmol, 1 eq.) was weighted in a dry vial, then degassed DCE (1.4 mL) was added. Then, $[Rh(CO)_2Cl]_2$ (1.4 mg, 0.004 mmol, 5 mol%) was added and the vial was then placed in an oven-dried stainless steel autoclave under argon, which was purged 3 times with CO then put under a pressure of 2 atm. It was stirred at room temperature for 18 h, then concentrated *in vacuo* and the crude was purified by silica flash chromatography (heptane:AcOEt 9:1 to 6:4). This afforded compound **2d** in 29% yield as a yellow foam (6.4 mg, 0.021 mmol), **3d** in 43% yield as a yellow oil (8.6 mg, 0.032 mmol) (**13** was detected in the crude mixture but was not isolated) .

Rf = 0.18 (pentane:AcOEt 85:15) **^{1}H NMR (400 MHz, CDCl$_3$):** δ 7.24 (s, 1H, H8), 6.51 (bs, 1H, H3), 5.05 (s, 2H, H20), 4.98, 4.84 (ABq, *J* = 14.4 Hz, 2H, H18), 3.12 (s, 3H, H14), 2.82 (d, *J* = 18.0 Hz, 1H, H15), 2.55-2.42 (m, 3H, H15', H23), 2.33 (m, 2H, H4, H5), 2.27 (m, 1H, H4'), 1.85 (m, 1H, H6), 1.74 (m, H6'), 1.37 (m, 1H, H5'), 1.11 (t, *J* = 7.2 Hz, 3H, H24) **^{13}C NMR (100 MHz, CDCl$_3$):** δ 183.6 (C25), 148.5 (C7), 147.8 (C22), 145.8 (C2), 142.1 (C16), 140.2 (21), 138.9 (C17), 129.8 (C3), 128.6 (C8), 79.2 (C1), 74.9 (C18), 74.5 (C20), 50.8 (C14), 41.6 (C15), 30.5 (C6), 25.7 (C4), 25.1 (C23), 17.2 (C5), 11.9 (C24) **HRMS :** (ESI)+ : Calculated for $[C_{19}H_{23}O_3]^+$: 299,1647, found : 299.16341 (Diff. : 2.47 ppm) **IR:** ν 2924, 2853, 2359, 2341, 1456, 1056, 669

3a. 4-ethyl-5-pentyl-1,3,7,8,9,10-hexahydro-9aH-fluoreno[1,2-c]furan-9aa-ol

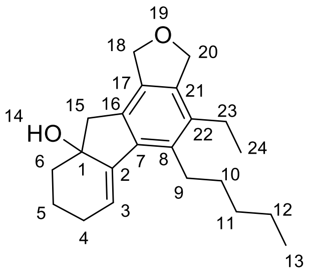

Chemical Formula: $C_{22}H_{30}O_2$
Molecular Weight: 326,48

See synthesis of **2a**.

Rf = 0.54 (pentane:AcOEt 85:15) **^{1}H NMR (400 MHz, CDCl$_3$):** δ 6.09 (bs, 1H, H3), 5.11 (s, 2H, H20), 5.09, 5.01 (ABq *J* = 11.8 Hz, 2H, H18), 2.86, 2.76 (ABq *J* = 17.0 Hz, 2H, H15), 2.75 (m, 2H, H9), 2.61 (m, 1H, H23), 2.51 (m, 1H, H23'), 2.41 (m, 1H, H4), 2.25 (m, 2H, H4', H6), 1.89 (m, 2H, H5), 1.81 (bs, 1H, H14), 1.66 (m, 1H, H6'), 1.57 (m, 2H, H10), 1.43 (m, 4H, H11, H12), 1.14 (t, *J* = 7.6 Hz, 3H, H24), 0.94 (t, *J* = 6.8 Hz, 3H, H13) **^{13}C NMR (100 MHz, CDCl$_3$):** δ 143.8 (C16), 137.9 (C21), 137.3 (C2), 136.4 (C8), 134.5 (C22), 133.3 (C17), 133.0 (C7), 123.6 (C3), 76.9 (C1), 73.4 (C18), 73.1 (C20), 44.6 (C15), 34.3 (C6), 32.3 (C11), 30.5 (C10), 29.1 (C9), 25.8 (C4), 23.1 (C23), 22.5 (C12), 17.6 (C5), 14.9 (C24), 14 .1 (C13) **HRMS :** (ESI)+ : Calculated for $[C_{22}H_{30}O_2Na]^+$: 349.2143, found : 349.21434 (Diff. : -1.64 ppm) **IR:** ν 3390, 2928, 2869, 2359, 2341, 1749, 1455, 1265, 1055, 922, 734

3c. 4-ethyl-9a-methoxy-5-pentyl-3,7,8,9,9a,10-hexahydro-1H-fluoreno[1,2-c]furan

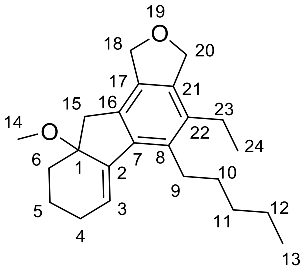

Chemical Formula: $C_{23}H_{32}O_2$
Molecular Weight: 340,51

See synthesis of **2c.**

Rf = 0.39 (pentane:AcOEt 85:15) **^{1}H NMR (400 MHz, CDCl$_3$):** δ 6.16 (bs, 1H, H3), 5.11 (s, 2H, H20), 5.09, 5.02 (ABq *J* = 12.0 Hz, 2H, H18), 3.07 (s, 3H, H14), 2.98 (d, *J* = 16.4 Hz, 1H, H15), 2.77 (m, 2H, H9), 2.64-2.47 (m, 3H, H15', H23), 2.43 (m, 1H, H4), 2.36 (m, H6), 2.24 (m, H4'), 1.90 (m, 1H, H5), 1.70 (m, 2H, H5', H10), 1.53-1.39 (m, 6H, H6', H10', H11, H12), 1.11 (t, *J* = 7.6 Hz, 3H, H24), 0.93 (t, *J* = 6.8 Hz, 3H, H13) **^{13}C NMR (100 MHz, CDCl$_3$):** δ 142.3 (C2), 138.5 (C7), 137.6 (C21), 135.5 (C8), 134.4 (C22), 132.8 (C17), 132.4 (C16), 124.7 (C3), 81.2 (C1), 73.4 (C18), 73.1 (C20), 50.7 (C14), 39.1 (C15), 32.3 (C11), 31.4 (C6), 30.2 (C10), 29.0 (C9), 25.8 (C4), 23.2 (C23), 22.5 (C12), 17.1 (C5), 14.9 (C24), 14.9 (C13) **HRMS :** (ESI)+ : Calculated for $[C_{23}H_{32}O_2Na]^+$: 363.4968, found : 363.22907 (Diff. : 1.14 ppm) **HRMS :** (ESI)+ : Calculated for $[C_{46}H_{64}O_4Na]^+$: 703.4702, found : 703.46867 (Diff. : 1.48 ppm) **IR:** ν 2927, 2870, 2359, 2341, 1455, 1075, 1051, 969, 908

3d. 4-ethyl-9a-methoxy-3,7,8,9,9a,10-hexahydro-1H-fluoreno[1,2-c]furan

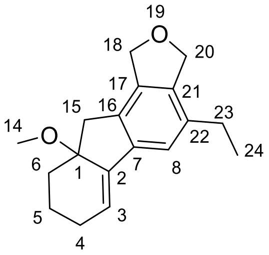

Chemical Formula: $C_{18}H_{22}O_2$
Molecular Weight: 270,37

See synthesis of **2d.**

Rf = 0.37 (pentane:AcOEt 85:15) **^{1}H NMR (400 MHz, CDCl$_3$):** δ 7.16 (s, 1H, H8), 6.18 (bs, 1H, H3), 5.10 (m, 3H, H20, H18), 5.03 (d, *J* = 12.4 Hz, 1H, H18'), 3.10 (s, 3H, H14), 3.04, 2.58 (ABq, *J* = 16.9 Hz, 2H, H15), 2.52 (m, 2H, H23), 2.36 (m, 2H, H4, H5), 2.23 (m, 1H, H4'), 1.94 (m, 1H, H6), 1.72 (m, 1H, H6'), 1.47 (td, *J* = 13.8, 3.1 Hz, 1H, H5'), 1.21 (t, *J* = 7.6 Hz, 3H, H24) **^{13}C NMR (100 MHz, CDCl$_3$):** δ 141.5 (C2), 140.4 (C7), 136.5 (C21), 135.6 (C22), 134.3 (C17), 131.4 (C16), 121.5 (C3), 117.5 (C8), 80.4 (C1), 72.4 (C18), 72.3 (C20), 50.0 (C14), 38.3 (C15), 30.5 (C6), 25.8 (C23), 24.3 (C4), 16.9 (C5), 13.8 (C24) **HRMS :** (ESI)+ : Calculated for $[C_{18}H_{22}O_2Na]^+$: 293.1517, found : 293.1509 (Diff. : 0.99 ppm) **IR:** ν 2934, 2359, 1755, 1455, 1333, 1049, 907, 727, 647

5. 2-iodocyclohex-2-en-1-one

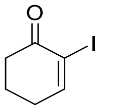

Chemical Formula: C_6H_7IO
Molecular Weight: 222,03

This compound was synthesised using a described procedure. Spectroscopic data for this intermediate match with those reported in the literature.[1]

6. 2-(hept-1-yn-1-yl)cyclohex-2-en-1-one

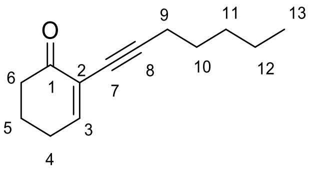

Chemical Formula: $C_{13}H_{18}O$
Molecular Weight: 190,29

Compound **6** was obtained following general procedure II using 2-iodocyclohexenone **5** (400 mg, 1.784 mmol, 1 eq.), CuI (13.6 mg, 0.036 mmol, 4 mol%), $PdCl_2(PPh_3)_2$ (25.1 mg, 0.071 mmol, 2 mol%), benzene (3.8 mL), Et_3N (1.14 mL, 8.21 mmol, 4.6 eq.), and 1-heptyne (0.47 mL, 3.567 mmol, 2 eq.). Work-up and purification afforded **6** (296.4 mg, 1.56 mmol) as a yellow oil in 87% yield.

Rf = 0.73 (pentane:AcOEt 8:2) **^{1}H NMR (400 MHz, CDCl$_3$):** δ 7.19 (t, *J* = 4.4 Hz, 1H, H3), 2.48 (t, *J* = 7.0 Hz, 2H, H9), 2.42 (m, 2H, H4), 2.36 (t, *J* = 7.0 Hz, 2H, H6), 2.01 (m, 2H, H5), 1.56 (m, 2H, H10), 1.35 (m, 4H, H11, H12), 0.90 (t, *J* = 7.0 Hz, 3H, H13) **^{13}C NMR (100 MHz, CDCl$_3$):** δ 196.3 (C1), 153.2 (C3), 125.8 (C2), 93.7 (C-8), 75.1 (C7), 38.4 (C6), 31.3 (C11), 28.6 (C10), 26.6 (C6), 22.7 (C4), 22.4 (C9), 19.7 (C5), 14.2 (C13) **HRMS:** (ESI)+ Calculated for $[C_{13}H_{19}O]^+$: 191.1435, found : 191.1422 (Diff. : 4.66 ppm) **IR:** ν 2929, 2860, 1688, 1455, 1427, 1351, 1149, 1118, 903.

7. 2-((trimethylsilyl)ethynyl)cyclohex-2-en-1-one

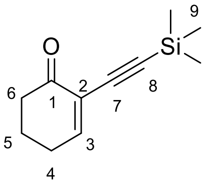

Chemical Formula: $C_{11}H_{16}OSi$
Molecular Weight: 192,33

Compound **7** was obtained following general procedure II using 2-iodocyclohexenone **5** (500 mg, 2.252 mmol, 1 eq.), CuI (17.2 mg, 0.090 mmol, 4 mol%), $PdCl_2(PPh_3)_2$ (31.7 mg, 0.045 mmol, 2 mol%), benzene (5 mL), Et_3N (1.5 mL, 10.79 mmol, 4.8 eq.), and trimethylsilylacetylene (0.64 mL, 4.504 mmol, 2 eq.). Work-up and purification afforded **7** (401.7 mg, 2.08 mmol) as a yellow oil in 93% yield. Spectroscopic data for this intermediate match with those reported in the literature.[2]

8. 2-(hept-1-yn-1-yl)-1-(prop-2-yn-1-yl)cyclohex-2-en-1-ol

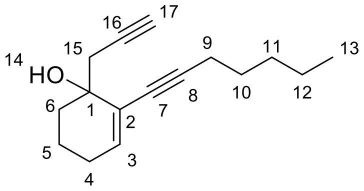

Chemical Formula: $C_{16}H_{22}O$
Molecular Weight: 230,35

Compound **8** was synthesised following general procedure III using **6** (200 mg, 1.051 mmol, 1eq.), magnesium turnings (46.0 mg, 1.892 mmol, 1.8 eq.), propargyl bromide 80% in toluene (0.17 mL, 1.595 mmol, 1.5 eq.), $HgCl_2$ (14.2 mg, 0.052 mmol, 5 mol%), I_2 (13.3 mg, 0.052 mmol, 5 mol%) and 0.5 mL dry Et_2O as the starting amount (total amount used: 3.5 mL). Work-up and purification afforded **8** as a yellow oil in 78% yield (188 mg, 28.9 mmol).

Rf = 0.6 (pentane:Et_2O 8:2) **^{1}H NMR (400 MHz, CDCl$_3$):** δ 6.16 (t, *J* = 4.4 Hz, 1H, H3), 2.77 (dd, *J* = 16.6, 2.6 Hz, 1H, H15), 2.55 (dd, *J* = 16.6, 2.6 Hz, 1H, H15'), 2.32 (t, *J* = 7.2 Hz, 2H, H9), 2.17 (s, 1H, H14), 2.12 (m, 2H, H4), 2.05 (dd, *J* = 2.6, 2.6 Hz, 1H, H17), 1.97 (m, 1H, H6), 1.87 (m, 1H, H6'), 1.73 (m, 1H, H5), 1.66 (m, 1H, H5'), 1.54 (m, 2H, H10), 1.38-1.35 (m, 4H, H11, H12), 0.90 (t, *J* = 7.2 Hz, 3H, H13) **^{13}C NMR (100 MHz, CDCl$_3$):** δ 137.0 (C3), 126.0 (C2), 91.8 (C8), 80.7 (C16), 77.7 (C7), 71.0 (C17), 70.3 (C1), 34.0 (C6), 31.5 (C15), 31.3 (C11), 28.6 (C10), 26.0 (C4), 22.3 (C12), 19.5 (C9), 18.7 (C5), 14.1 (C13) **HRMS :** (ESI)+ : Calculated for $[C_{16}H_{22}ONa]^+$: 253.1490, found : 253.1553 (Diff. : 4.35 ppm) **IR:** ν 3444, 3310, 2930, 2860, 1455, 1472, 1168, 1087, 989, 950, 816, 698, 632, 513

9. 1-(prop-2-yn-1-yl)-2-((trimethylsilyl)ethynyl)cyclohex-2-en-1-ol

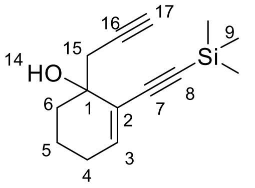

Chemical Formula: $C_{14}H_{20}OSi$
Molecular Weight: 232,3980

Compound **9** was synthesised following general procedure III using **7** (1.496 g, 7.778 mmol, 1eq.), magnesium turnings (0.340 g, 14 mmol, 1.8 eq.), propargyl bromide 80% in toluene (1.26 mL, 11.67 mmol, 1.5 eq.), $HgCl_2$ (0.105 g, 0.389 mmol, 5 mol%), I_2 (98.7 mg, 0.389 mmol, 5 mol%) and 5 mL dry Et_2O as the starting amount (total amount used: 35 mL). Work-up and purification afforded **9** as a yellow oil quantitatively (188 mg, 28.9 mmol).

Rf = 0.79 (pentane:AcOEt 9:1) **^{1}H NMR (400 MHz, CDCl$_3$):** δ 6.32 (t, *J* = 4.0 Hz, 1H, H3), 2.80 (dd, *J* = 16.6, 2.6 Hz, 2H, H15), 2.55 (dd, *J* = 16.6, 2.6 Hz, 2H, H15'), 2.21-2.09 (m, 2H, H4), 2.17 (t, *J* = 2.6 Hz, 1H, H17), 1.92 (m, 2H, H6), 1.74 (m, 1H, H5), 1.67 (m, 1H, H5'), 1.61 (bs, 1H, H14), 0.19 (s, 9H, H9) **^{13}C NMR (100 MHz, CDCl$_3$):** δ 139.8 (C3), 125.9 (C2), 102.4 (C8), 95.9 (C16), 80.5 (C7), 71.2 (C17), 69.9 (C1), 33.9 (C6), 31.5 (C15), 26.1 (C4), 18.5 (C5), 0.2 (C9) **HRMS :** (ESI)+ : Calculated for $[C_{14}H_{21}OSi]^+$: 233.4060, found : 233.1354 (Diff. : 0.68 ppm) **IR:** ν3427, 3290, 2953, 2142, 1248, 1161, 989, 837, 758, 696, 620

10. 2-(hept-1-yn-1-yl)-1-(4-hydroxybut-2-yn-1-yl)cyclohex-2-en-1-ol

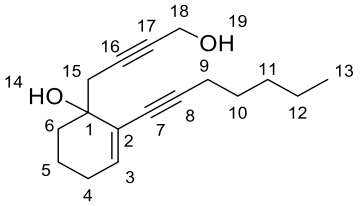

Chemical Formula: $C_{17}H_{24}O_2$
Molecular Weight: 260,38

10 was synthesised following general procedure IV using **8** (276.5 mg, 1.2 mmol, 1 eq.), paraformaldehyde (113.8 mg, 1.2 mmol, 1 eq.), EtMgBr 40% in 2-methylTHF (1.75 mL, 6.002 mmol, 5 eq.) and THF (11.6 mL). Work-up and purification afforded **10** as a clear oil in 74% yield (230.2 mg, 0.884 mmol).

Rf = 0.12 (pentane:AcOEt 8:2) **^{1}H NMR (400 MHz, CDCl$_3$):** δ 6.14 (t, *J* = 4.0 Hz, 1H, H3), 4.26 (bs, 2H, H18), 2.84, 2.53 (ABq, *J* = 16.6 Hz, 2H, H15), 2.31 (m, 3H, H9, H19), 2 .10 (m, 2H, H4), 2.01 (bs, 1H, H14), 195-186 (m, 2H, H6), 1.73 (m, 1H, H5), 1.63 (m, 1H, H5'), 1.54 (m, 2H, H10), 1.39-1.31 (m, 4H, H11, H12), 0.90 (t, *J* = 6.8 Hz, 3H, H13) **^{13}C NMR (100 MHz, CDCl$_3$):** δ 136.8 (C3), 126.1 (C2), 91.8 (C8), 82.4 (C16), 81.1 (C17), 77.6 (C7), 70.3 (C1), 51.4 (C18), 33.9 (C6), 31.6 (C11), 31.2 (C15), 28.5 (C10), 25.9 (C4), 22.2 (C12), 19.3 (C9), 18.7 (C5), 14.0 (C13)

11. 1-(4-hydroxybut-2-yn-1-yl)-2-((trimethylsilyl)ethynyl)cyclohex-2-en-1-ol

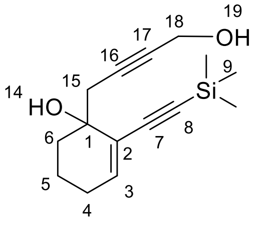

Chemical Formula: $C_{15}H_{22}O_2Si$
Molecular Weight: 262,42

11 was synthesised following general procedure IV using **9** (250 mg, 1.076 mmol, 1 eq.), paraformaldehyde (102 mg, 1.076 mmol, 1 eq.), EtMgBr 40% in 2-methylTHF (1.57 mL, 5.379 mmol, 5 eq.) and THF (10.8 mL). Work-up and purification afforded **11** as a clear oil in 83% yield (233.2 mg, 0.889 mmol).

Rf = 0.13 (pentane:AcOEt 8:2) **^{1}H NMR (400 MHz, CDCl$_3$):** δ 6.31 (t, *J* = 4.0 Hz, 1H, H3), 4.28 (bs, 2H, H18), 2.87, 2.54 (ABq *J* = 16.6 Hz, 2H, H15), 2.27 (bd, *J* = 10.2 Hz, 1H, H19), 2.15 (m, 2H, H4), 1.90 (m, 2H, H6), 1.76 (m, 1H, H5), 1.63 (m, 1H, H5'), 1.62 (bs, 1H, H14), 0.19 (s, 9H, H9) **^{13}C NMR (100 MHz, CDCl$_3$):** δ 139.6 (C3), 125.9 (C2), 102.3 (C8), 95.8 (C16), 82.2 (C7), 81.3 (C17), 69.9 (C1), 51.4 (C18), 33.8 (C6), 31.5 (C15), 26.0 (C4), 18.5 (C5), 0.0 (C9).

12. 4-ethyl-5-pentyl-3,7,8,9-tetrahydro-1H-fluoreno[1,2-c]furan

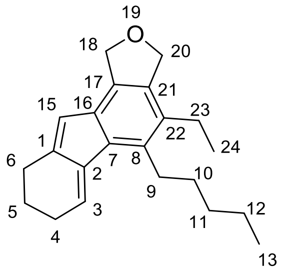

Chemical Formula: $C_{22}H_{28}O$
Molecular Weight: 308,47

See synthesis of **2a**.

Rf = 0.8 (pentane:AcOEt 85:15) **^{1}H NMR (400 MHz, $CDCl_3$):** δ 6.92 (bs, 1H, H3), 6.24 (s, 1H, H15), 5.15 (s, 2H, H20), 5.13 (s, 2H, H18), 2.84 (m, 2H, H9), 2.67 (m, 2H, H6), 2.54 (m, 4H, H4, H23), 1.86 (m, 2H, H5), 1.61 (m, 2H, H10), 1.43 (m, 4H, H11, H12), 1.14 (t, *J* = 7.6 Hz, 3H, H24), 0.94 (t, *J* = 6.8 Hz, 3H, H13) **^{13}C NMR (100 MHz, $CDCl_3$):** δ 140.6 (C2), 139.9 (C1), 137.5 (C21), 135.6 (C8), 134.5 (C16), 133.3 (C3), 131.5 (C22), 131.2 (C7), 127.9 (C17), 120.5 (C15), 73.4 (C18), 73.0 (C20), 32.6 (C11), 29.3 (C10), 28.8 (C9), 27.2 (C4), 25.6 (C6), 24.1 (C5), 23.2 (C23), 22.6 (C12), 15.0 (C24), 14.1 (C13) **HRMS :** (ESI)+ : Calculated for $[C_{22}H_{29}O]^+$: 309.2218 found : 309.22104 (Diff. : 0.83 ppm) **IR:** ν 2925, 2854, 1632, 1462, 1386, 1051, 906

Spectras

1a

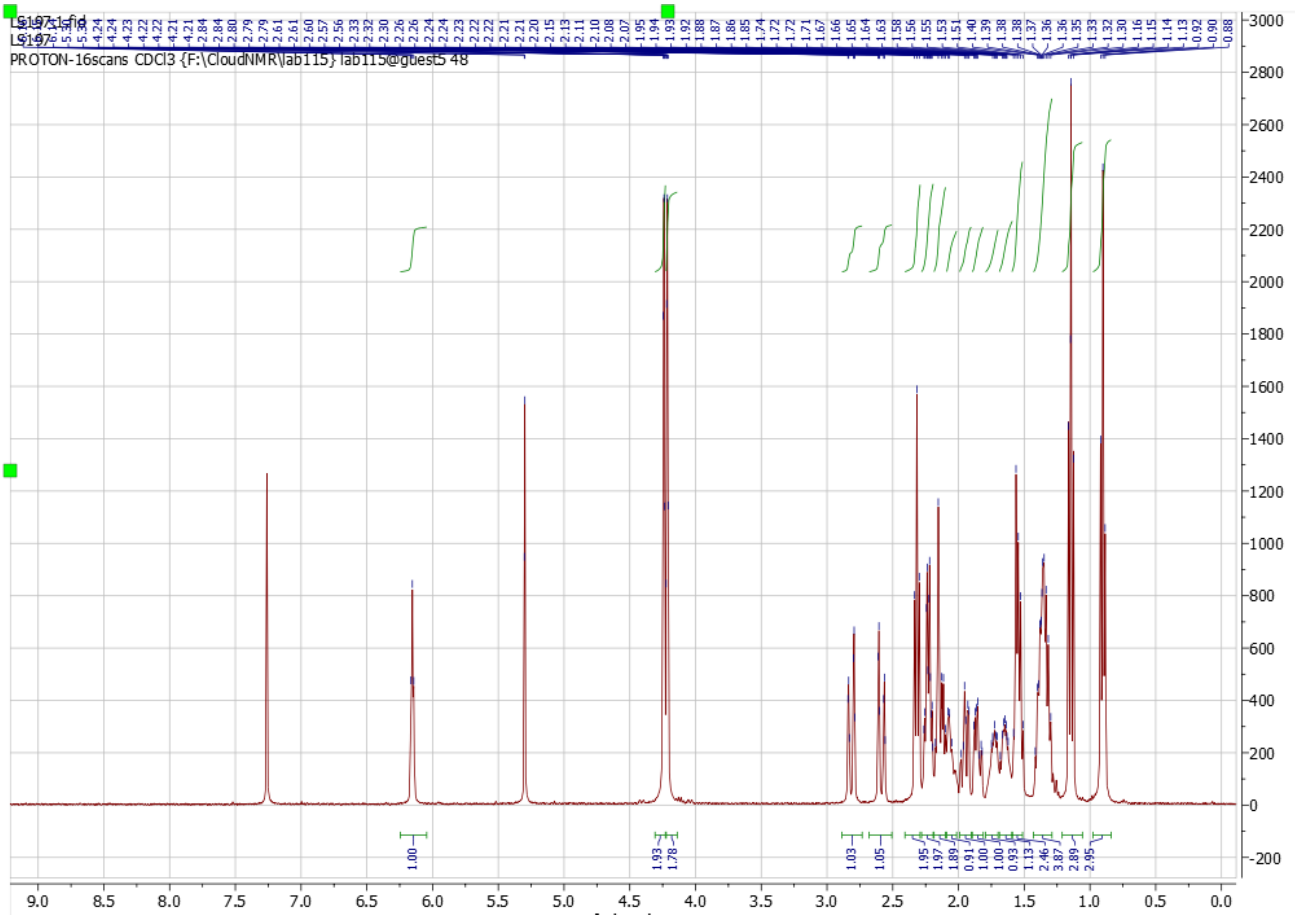

LS197.1.fid
LS197
PROTON-16scans CDCl3 {F:\CloudNMR\lab115} lab115@guest5 48

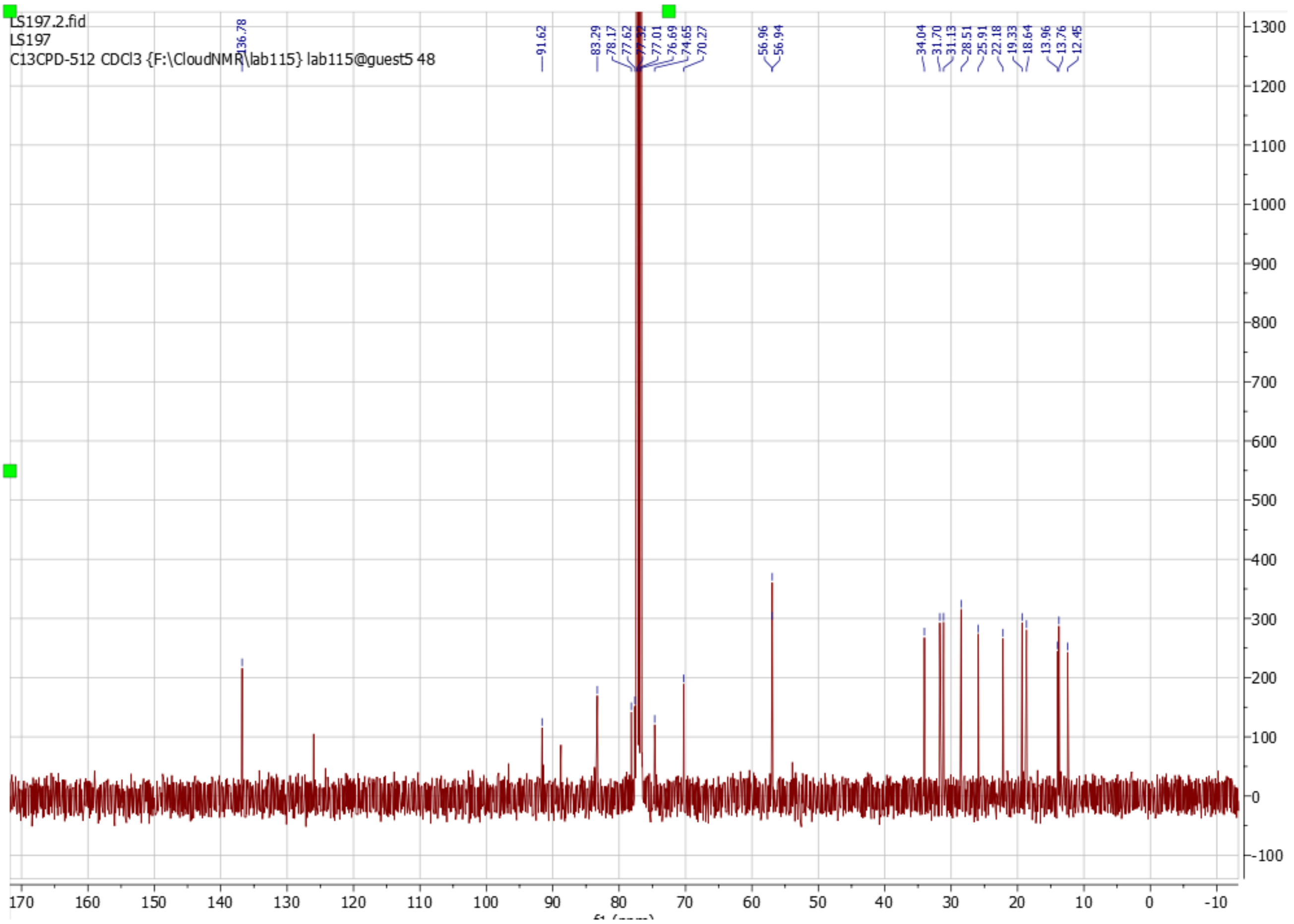

LS197.2.fid
LS197
C13CPD-512 CDCl3 {F:\CloudNMR\lab115} lab115@guest5 48

1b

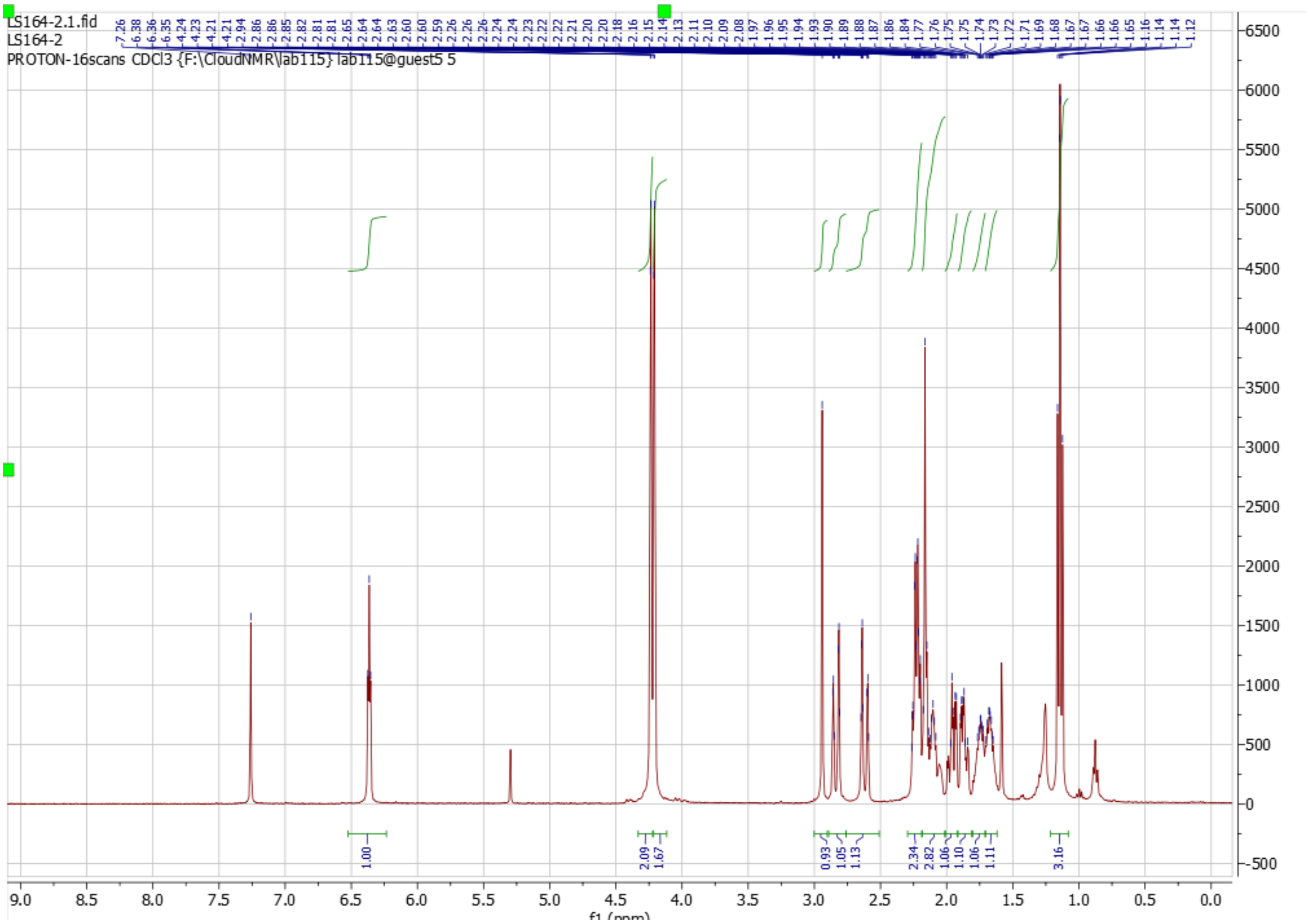
LS164-2.1.fid
LS164-2
PROTON-16scans CDCl3 {F:\CloudNMR\lab115} lab115@guest5 5
f1 (ppm)

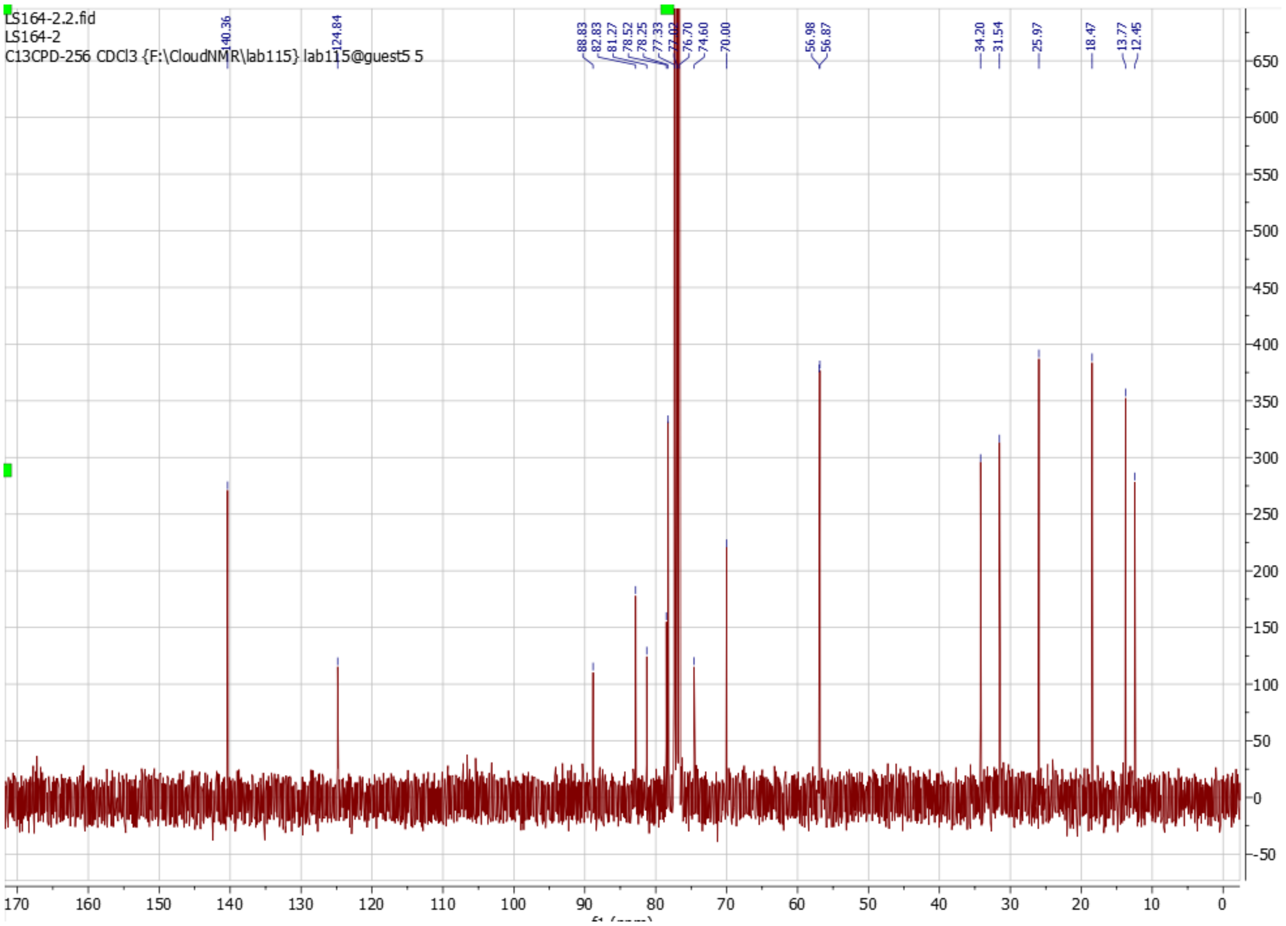
LS164-2.2.fid
LS164-2
C13CPD-256 CDCl3 {F:\CloudNMR\lab115} lab115@guest5 5
140.36
124.84
88.83
82.83
81.27
78.52
78.25
77.33
76.70
74.60
70.00
56.98
56.87
34.20
31.54
25.97
18.47
13.77
12.45

1c

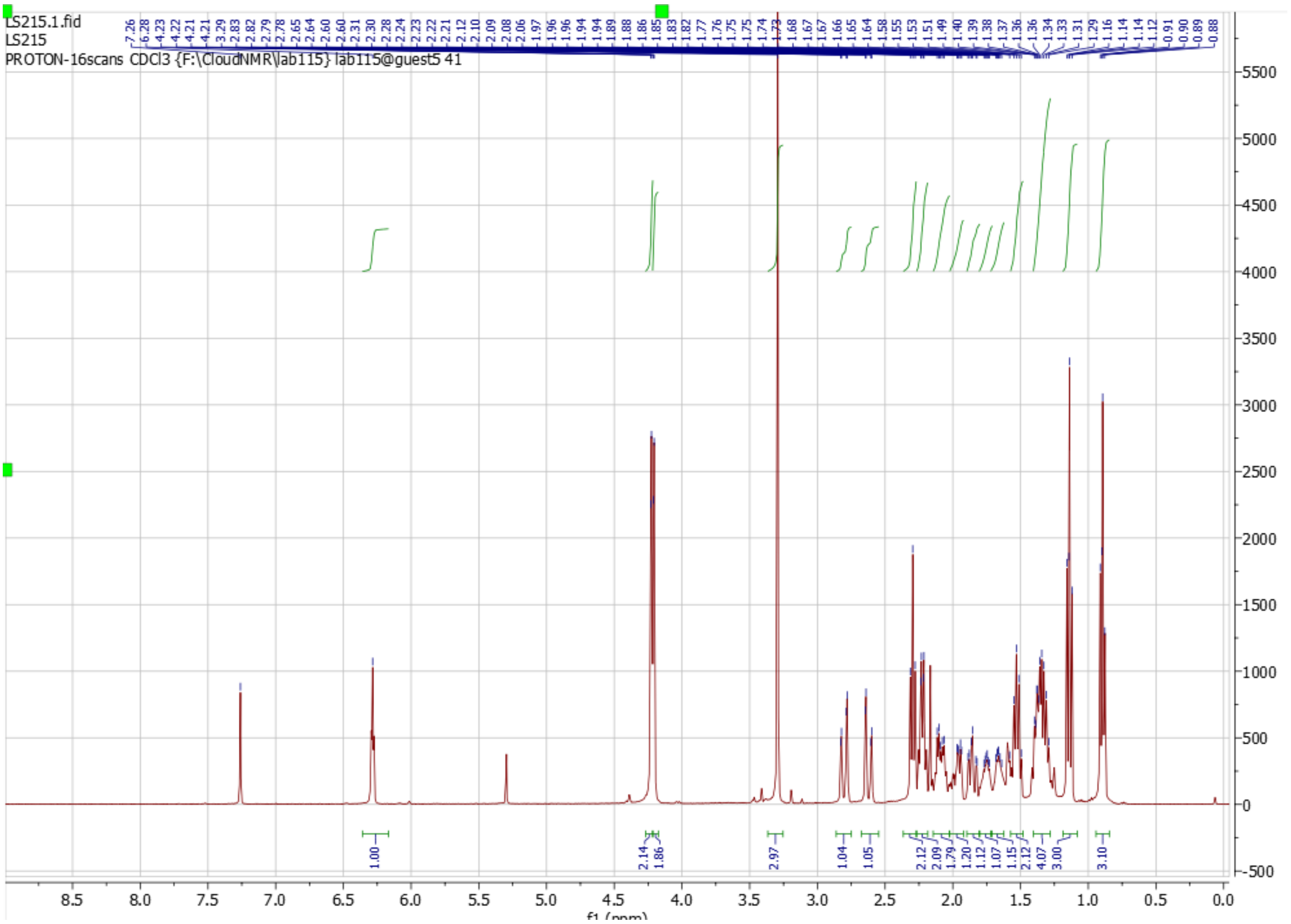
LS215.1.fid
LS215
PROTON-16scans CDCl3 {F:\CloudNMR\lab115} lab115@guest5 41
f1 (ppm)

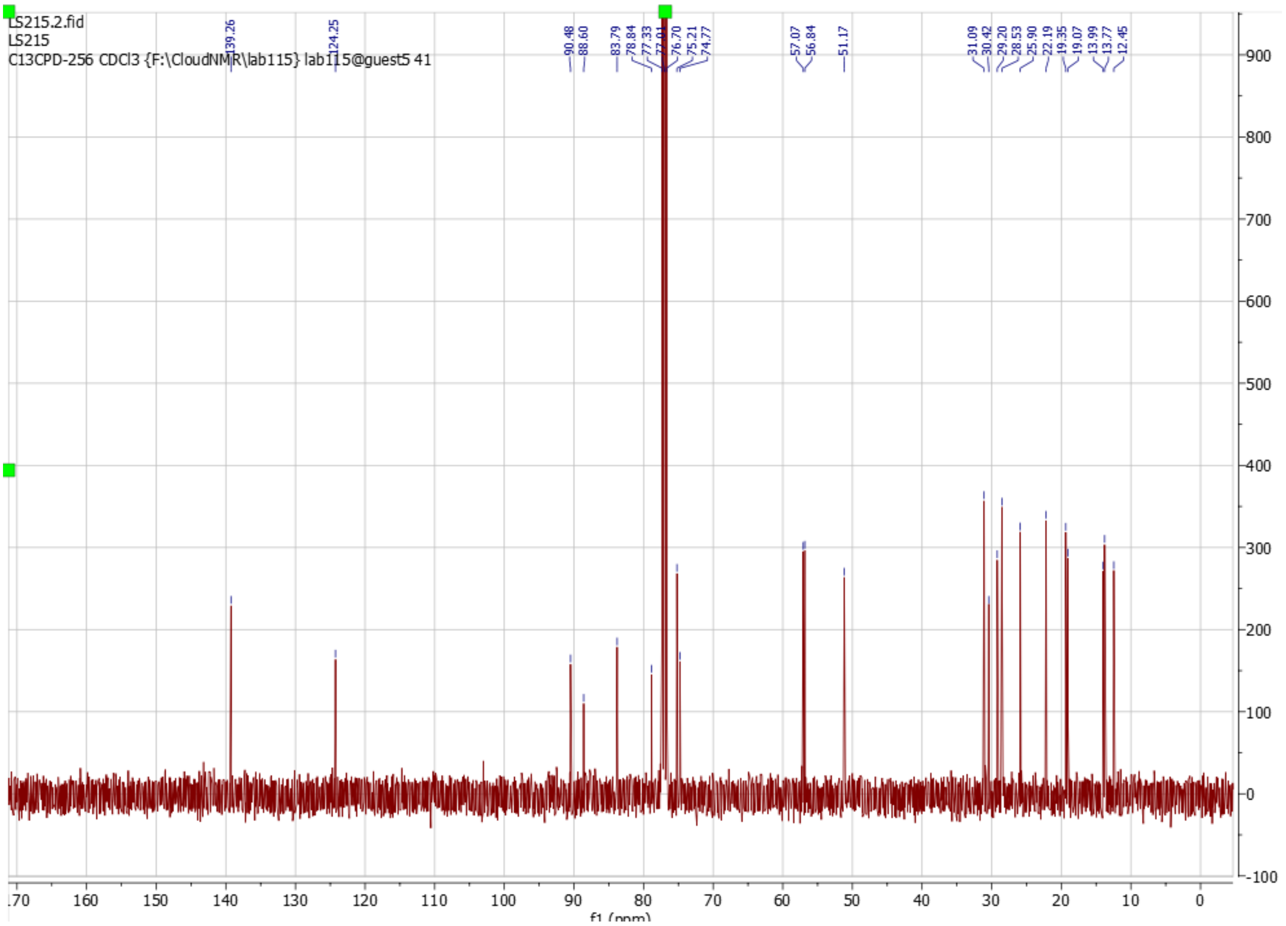
LS215.2.fid
LS215
C13CPD-256 CDCl3 {F:\CloudNMR\lab115} lab115@guest5 41
f1 (ppm)

1d

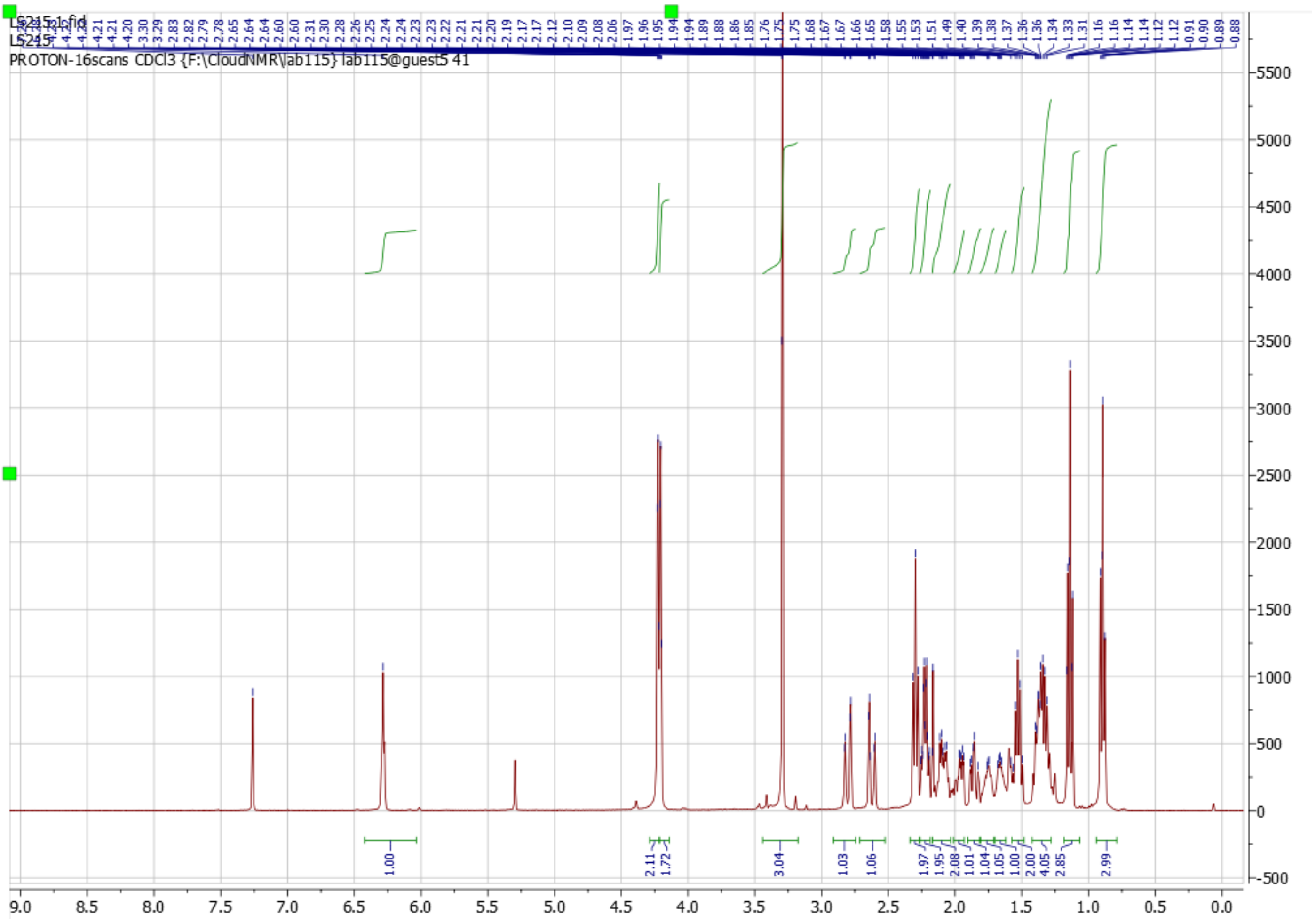
LS215.1.fid
LS215
PROTON-16scans CDCl3 {F:\CloudNMR\lab115} lab115@guest5 41

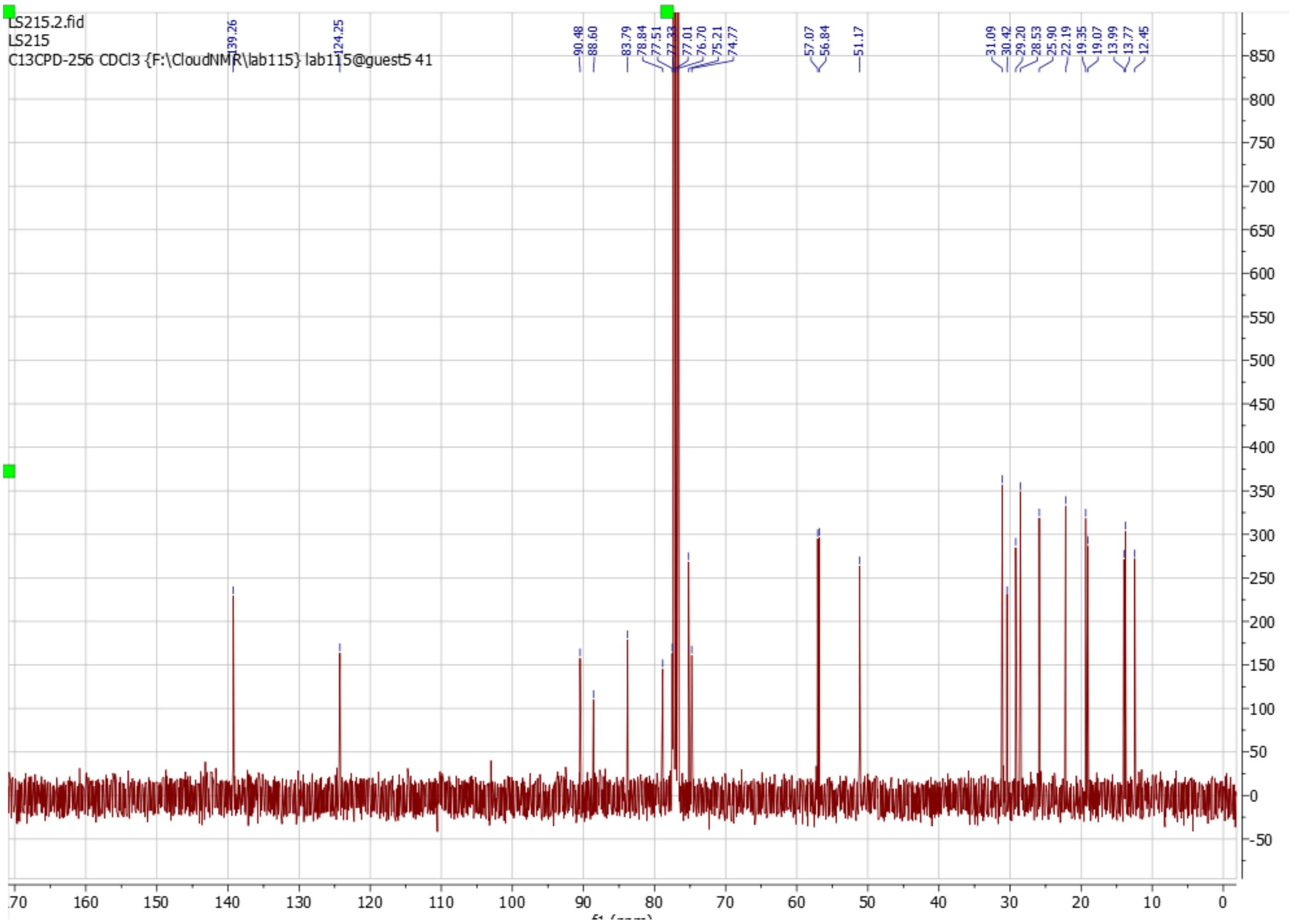
LS215.2.fid
LS215
C13CPD-256 CDCl3 {F:\CloudNMR\lab115} lab115@guest5 41
139.26
124.25
90.48
88.60
83.79
78.84
77.51
77.33
77.01
76.70
75.21
74.77
57.07
56.84
51.17
31.09
30.42
29.20
28.53
25.90
22.19
19.35
19.07
13.99
13.77
12.45

2a

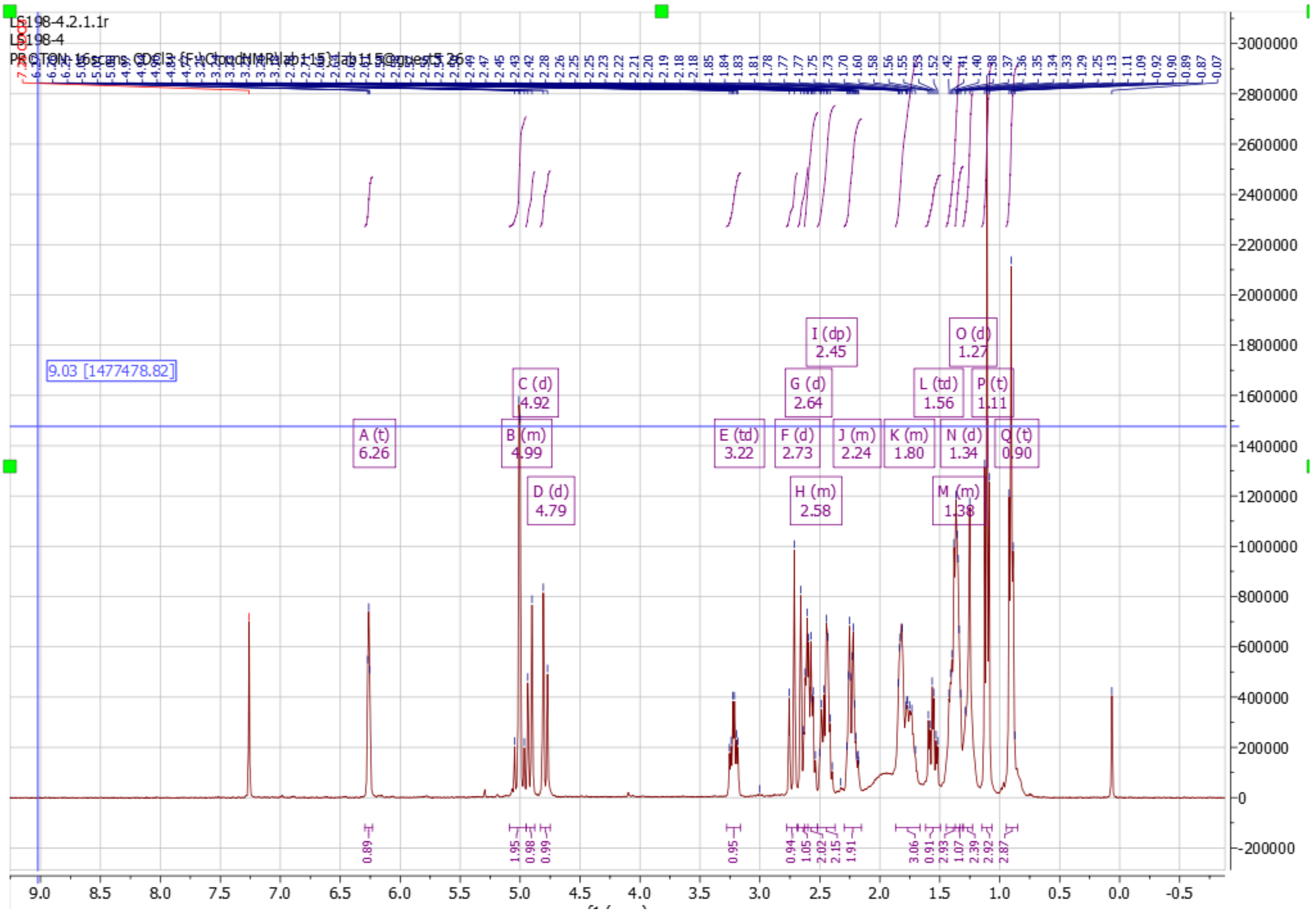
LS198-4.2.1.1r
LS198-4
PROTON 16scans CDCl3 {F:\CloudNMR\lab115} lab115@guest5 26
A (t) 6.26
B (m) 4.99
C (d) 4.92
D (d) 4.79
E (td) 3.22
F (d) 2.73
G (d) 2.64
H (m) 2.58
I (dp) 2.45
J (m) 2.24
K (m) 1.80
L (td) 1.56
M (m) 1.38
N (d) 1.34
O (d) 1.27
P (t) 1.11
Q (t) 0.90
9.03 [1477478.82]

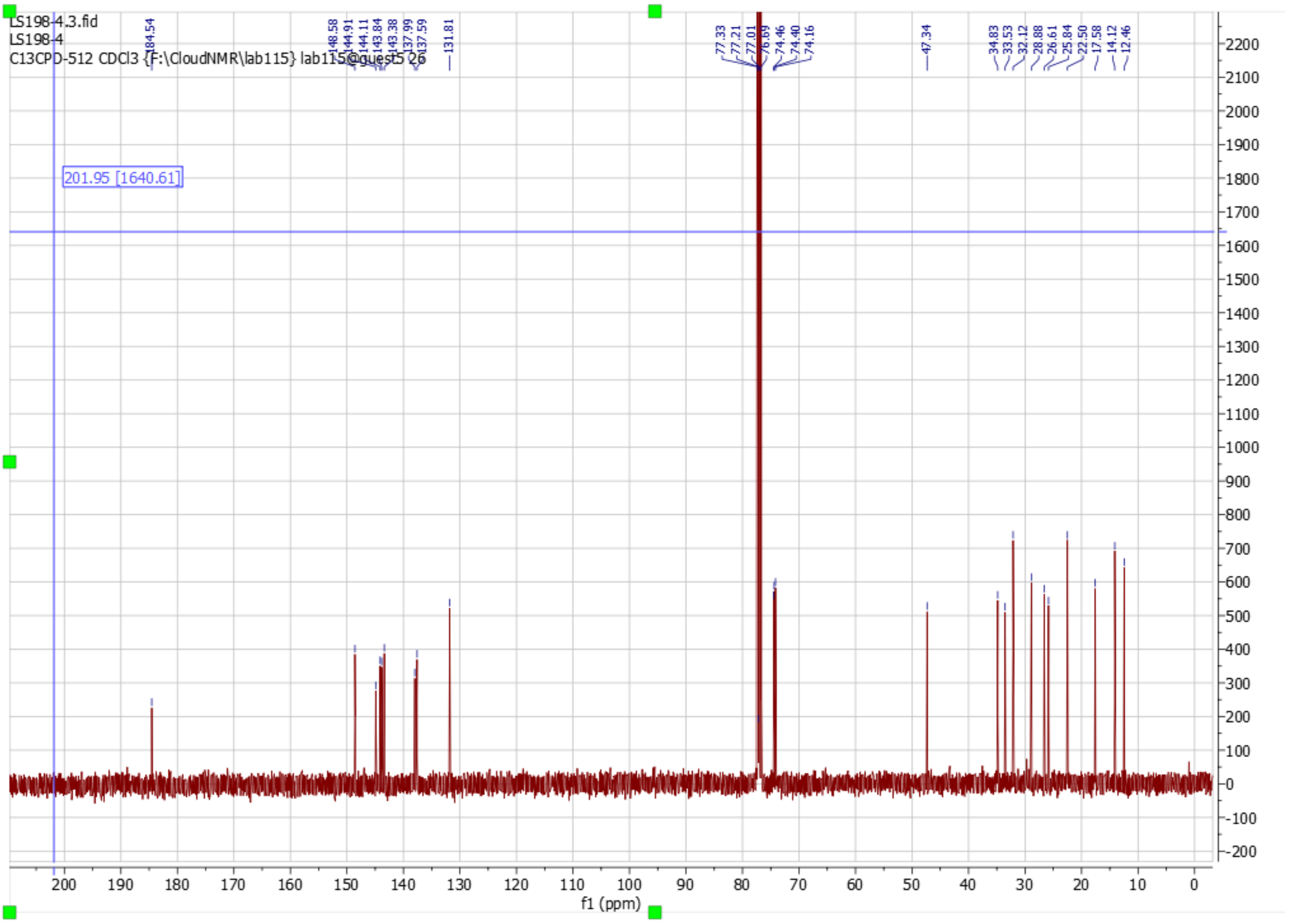
LS198-4.3.fid
LS198-4
C13CPD-512 CDCl3 {F:\CloudNMR\lab115} lab115@guest5 26
184.54
148.58
144.91
144.11
143.84
143.38
137.99
137.59
131.81
77.33
77.21
77.01
76.69
74.46
74.40
74.16
47.34
34.83
33.53
32.12
28.88
26.61
25.84
22.50
17.58
14.12
12.46
201.95 [1640.61]
f1 (ppm)

2c

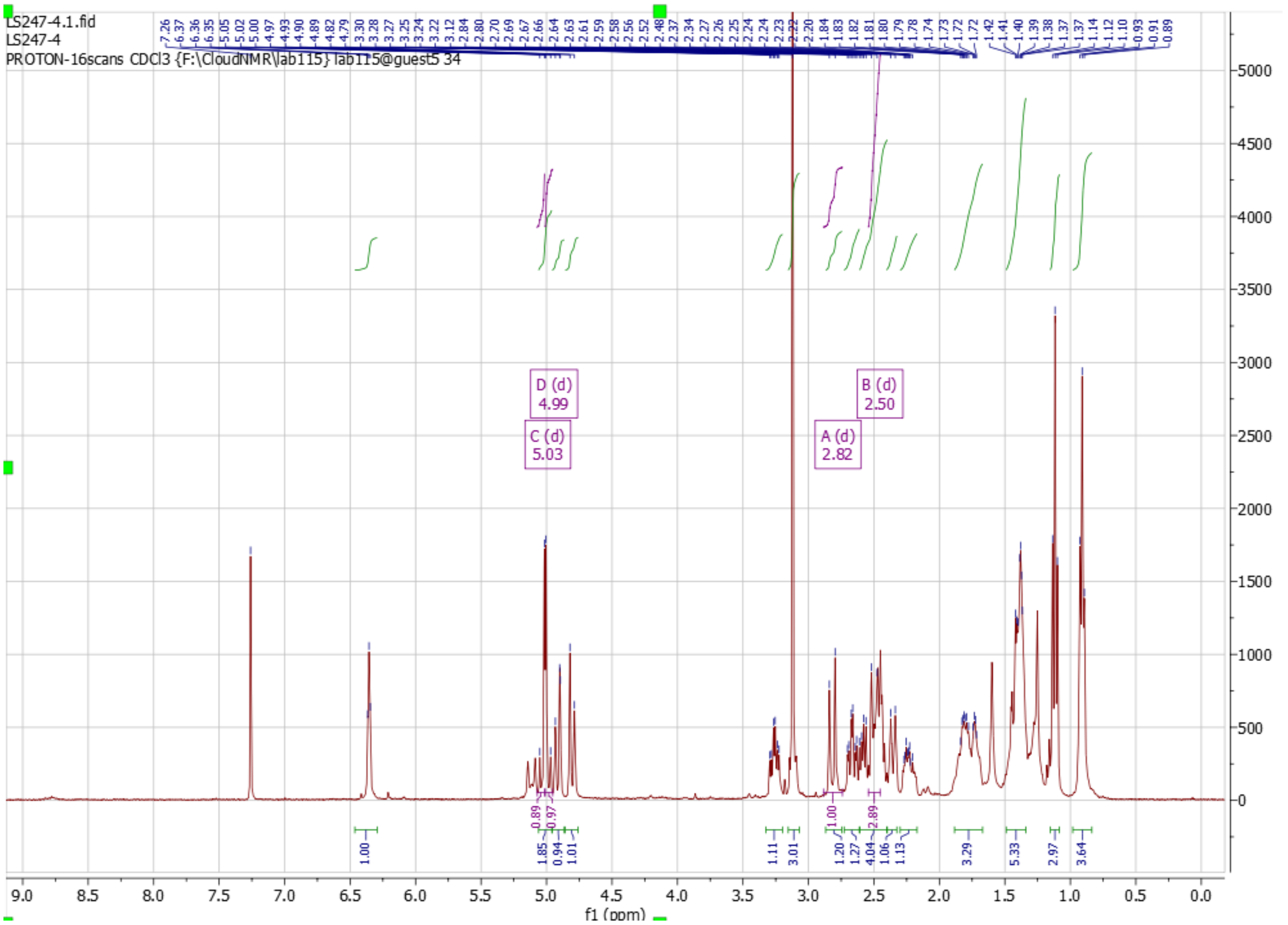

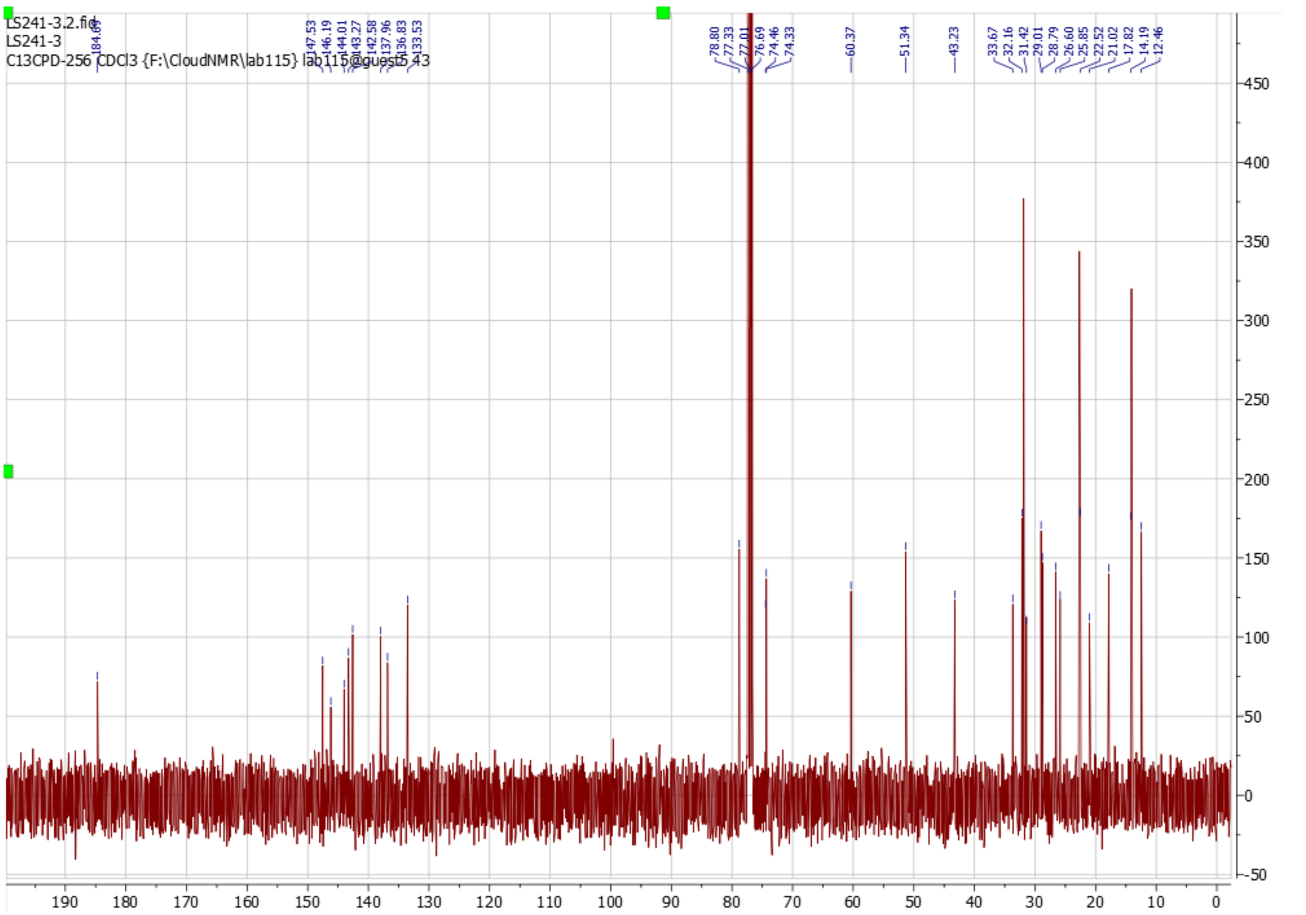

2d

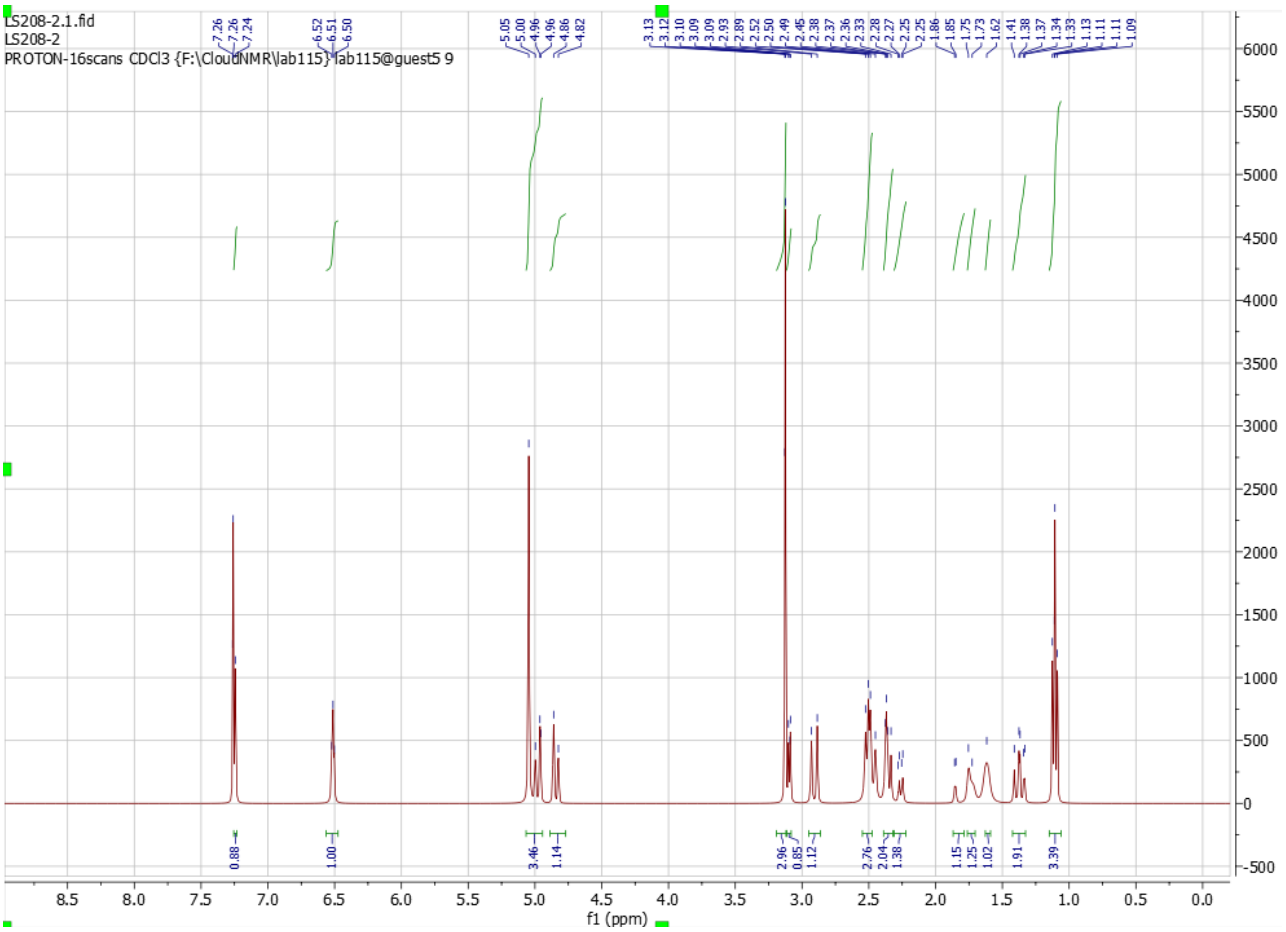
LS208-2.1.fid
LS208-2
PROTON-16scans CDCl3 {F:\CloudNMR\lab115} lab115@guest5 9
f1 (ppm)

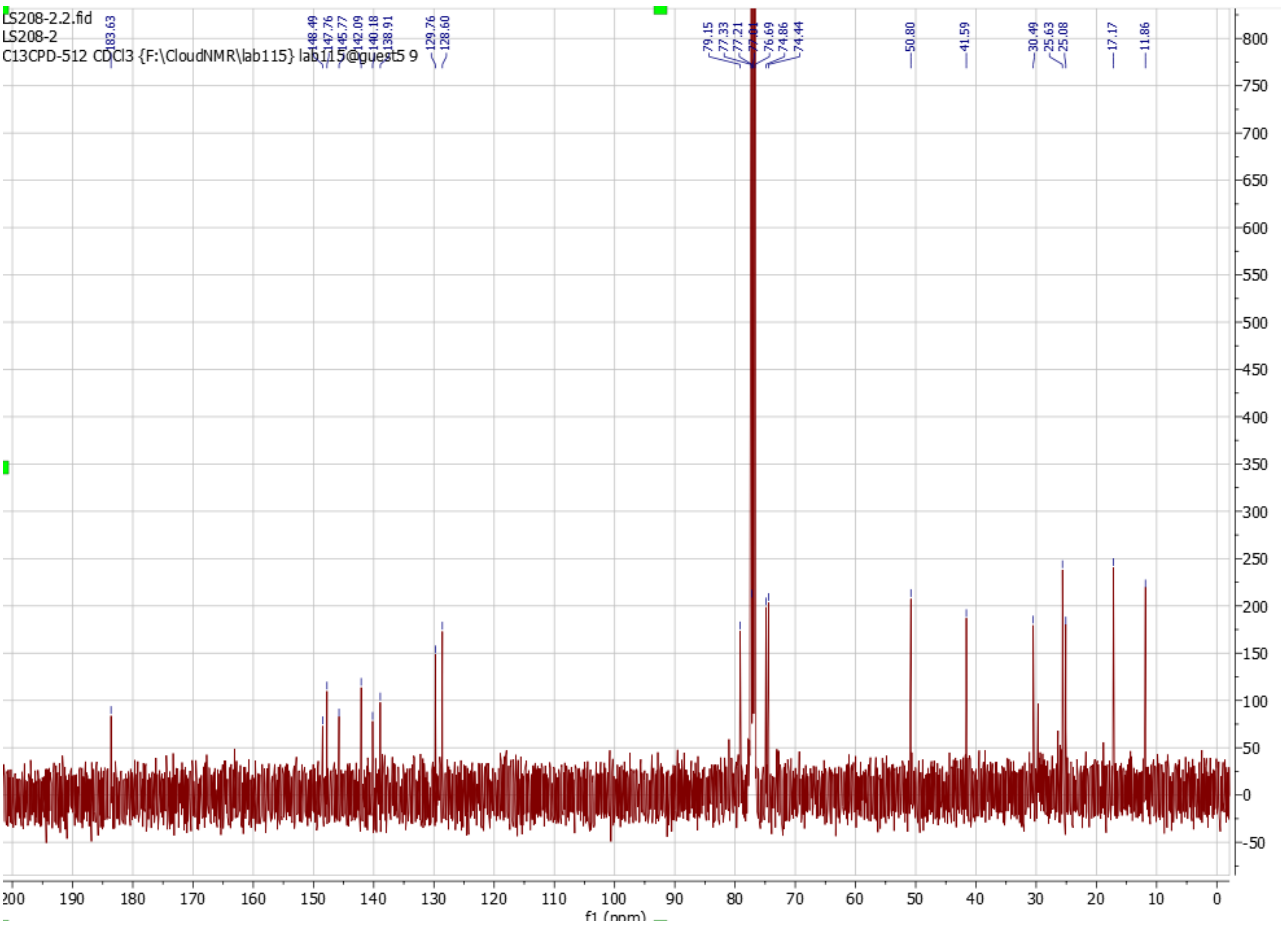
LS208-2.2.fid
LS208-2
C13CPD-512 CDCl3 {F:\CloudNMR\lab115} lab115@guest5 9
f1 (ppm)

3a

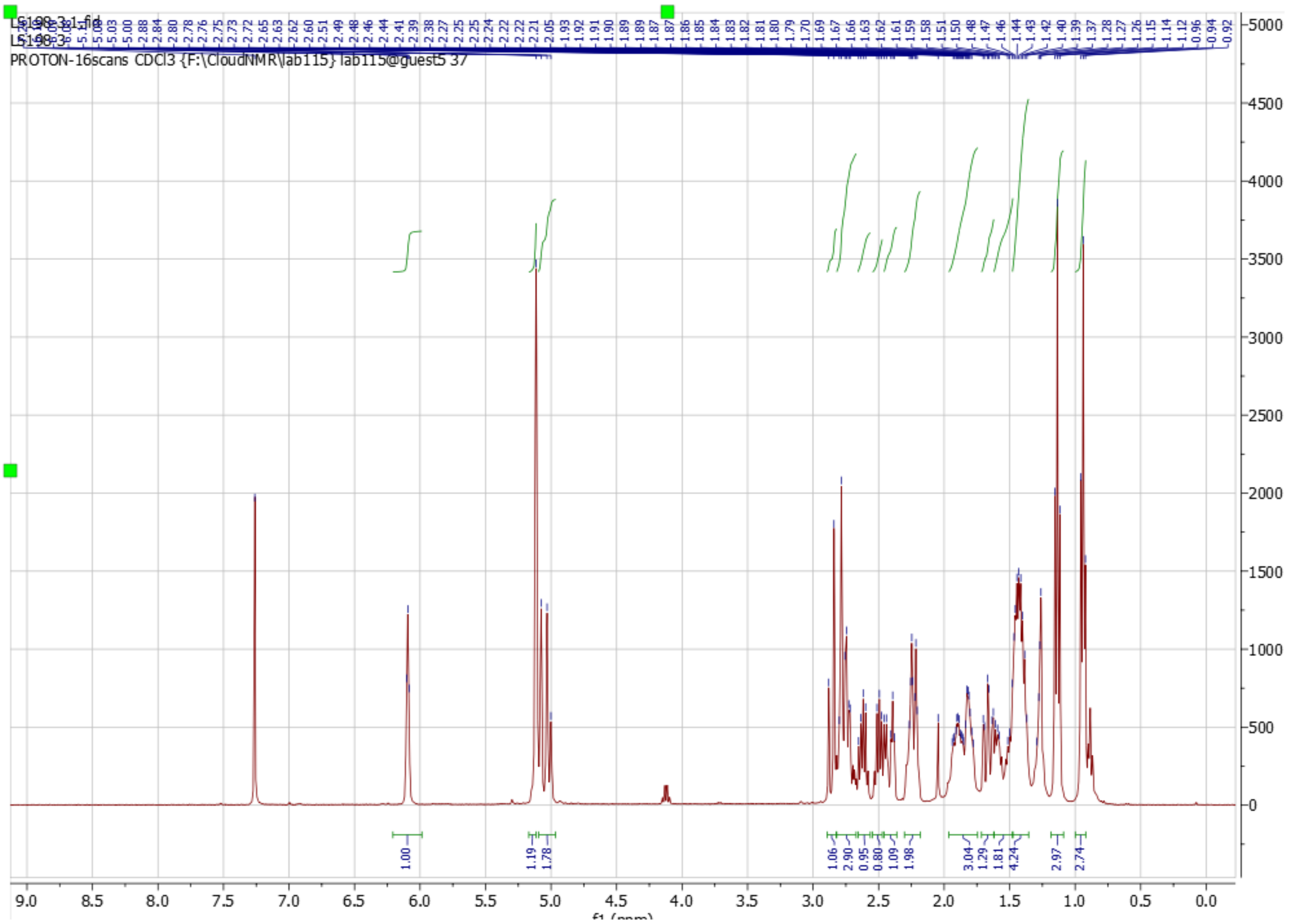

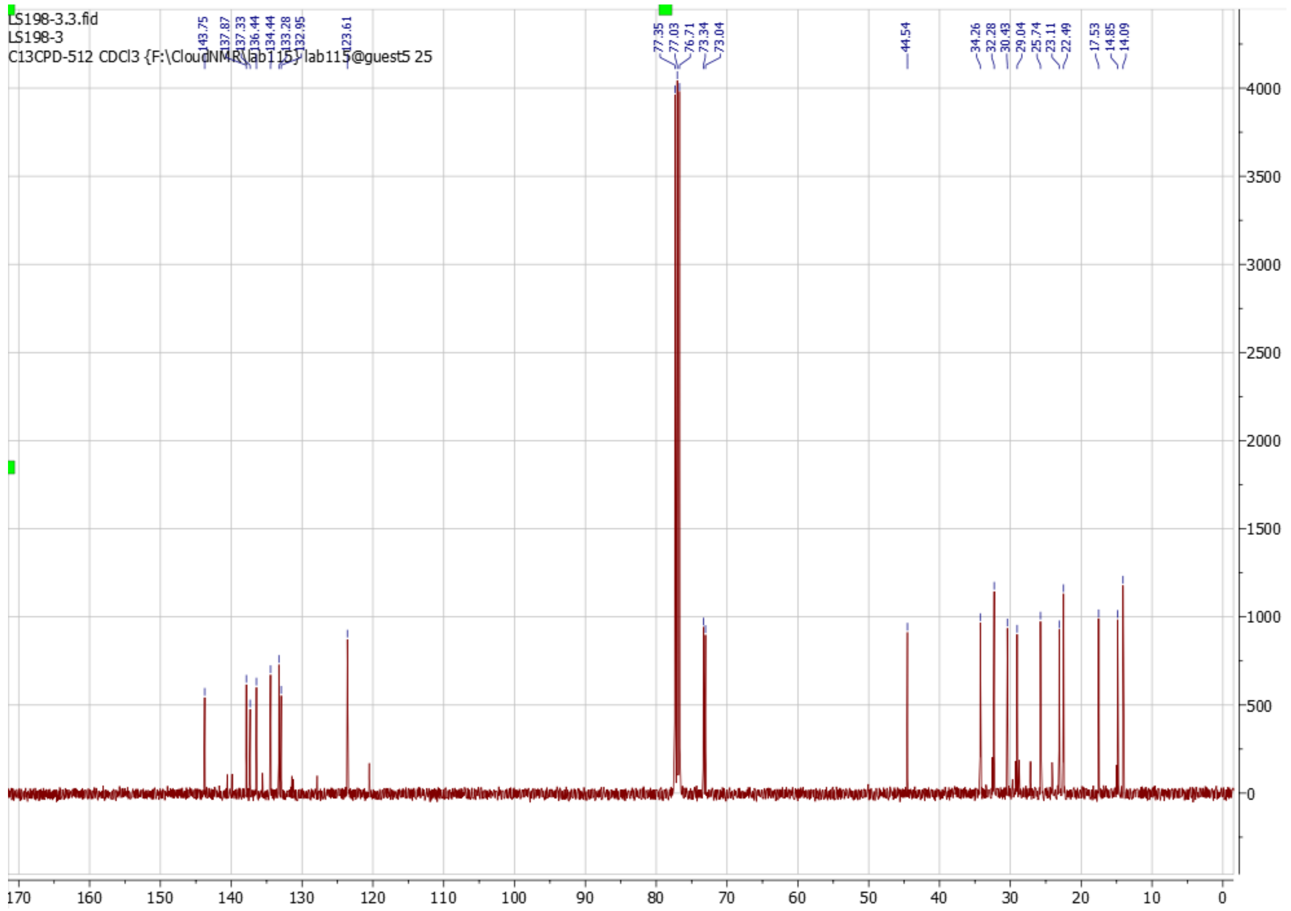

3c

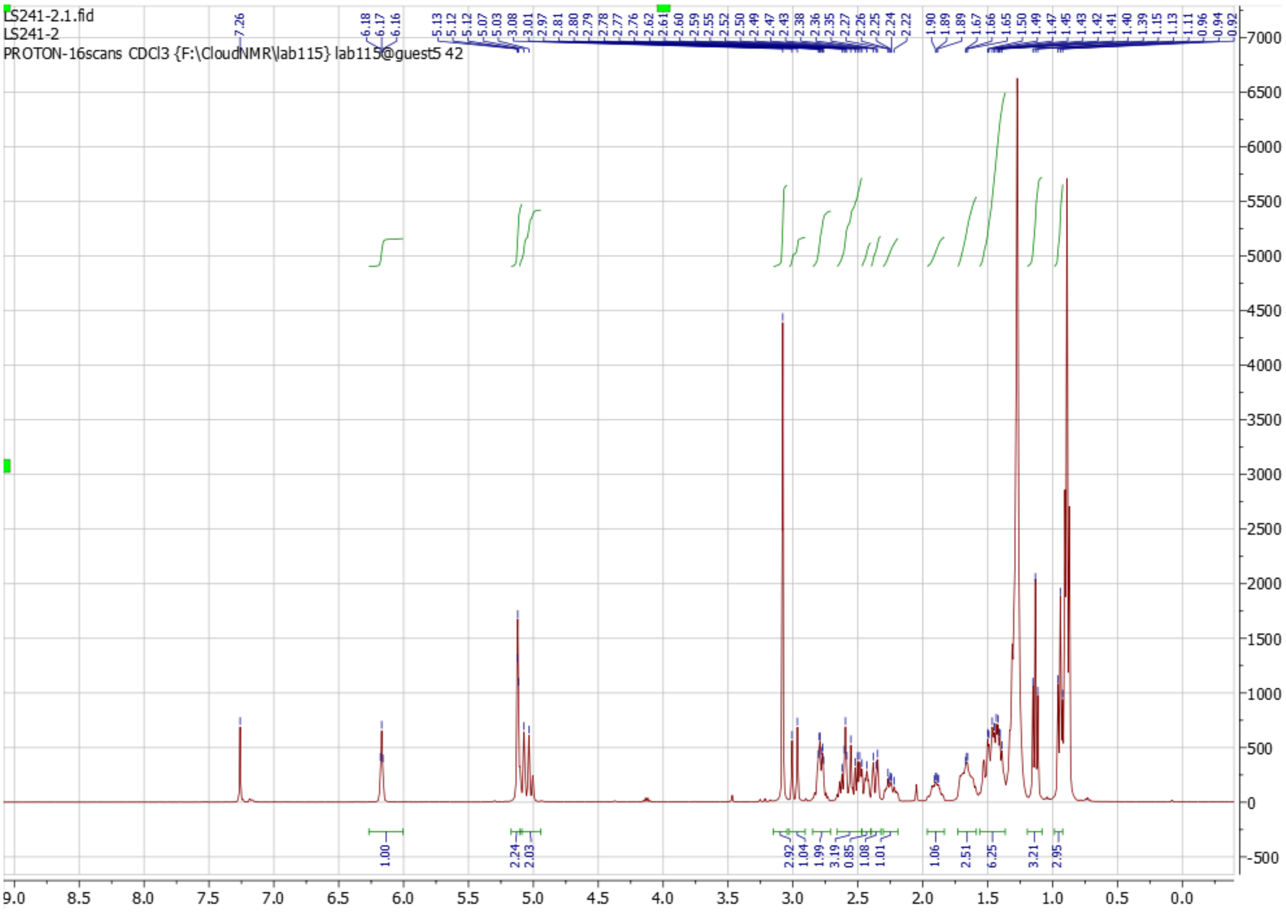
LS241-2.1.fid
LS241-2
PROTON-16scans CDCl3 {F:\CloudNMR\lab115} lab115@guest5 42

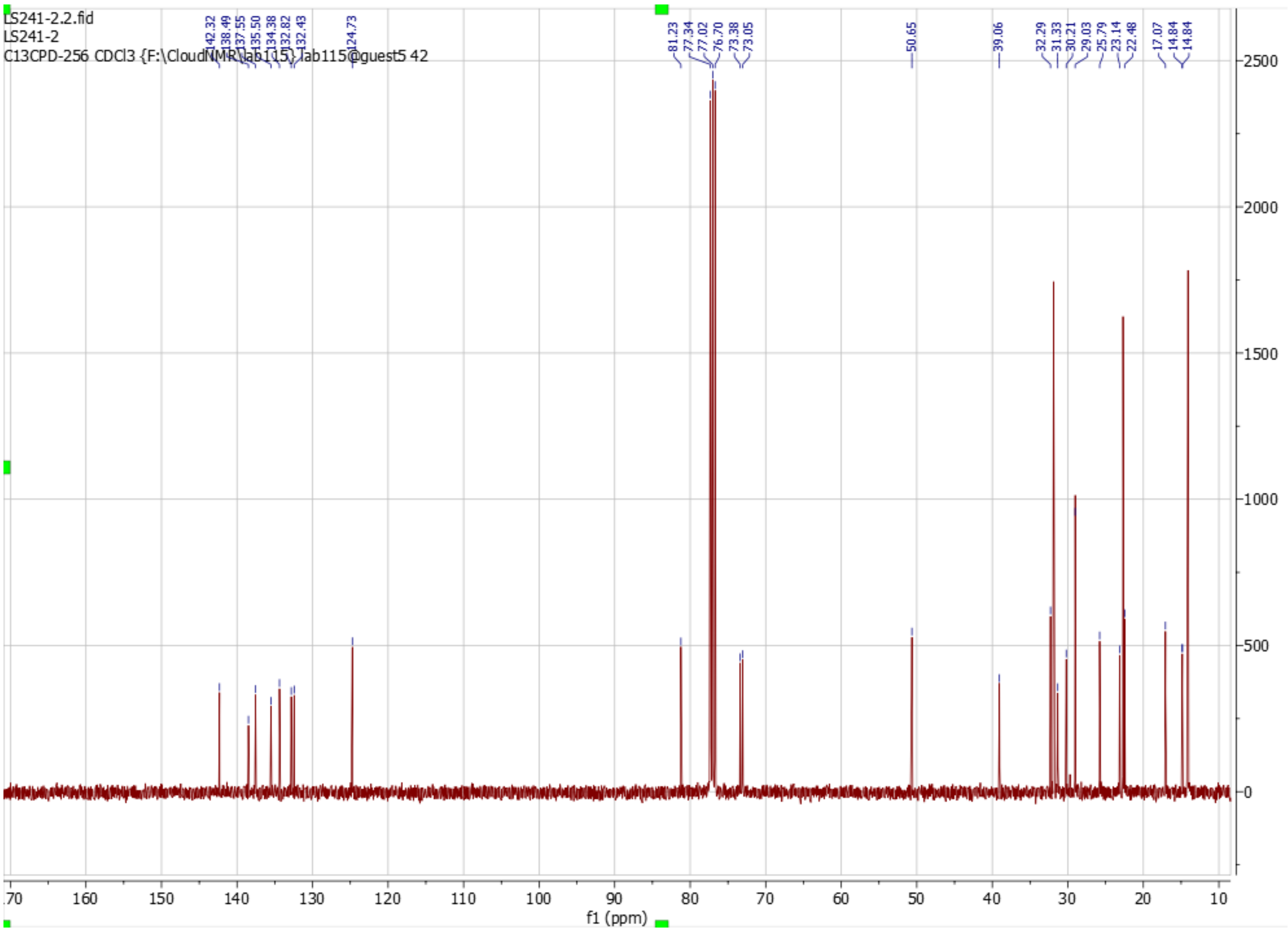
LS241-2.2.fid
LS241-2
C13CPD-256 CDCl3 {F:\CloudNMR\lab115} lab115@guest5 42
f1 (ppm)

3d

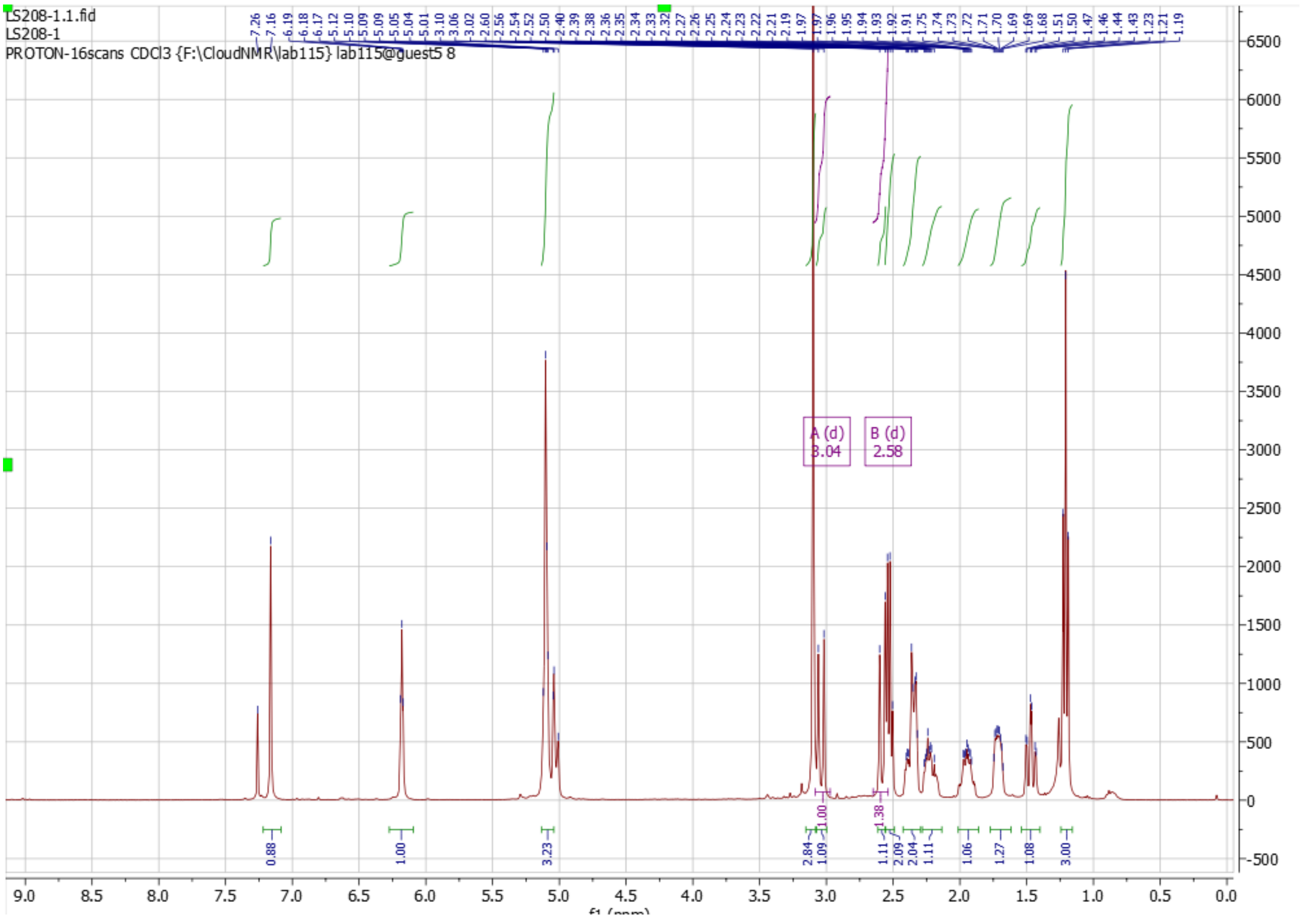

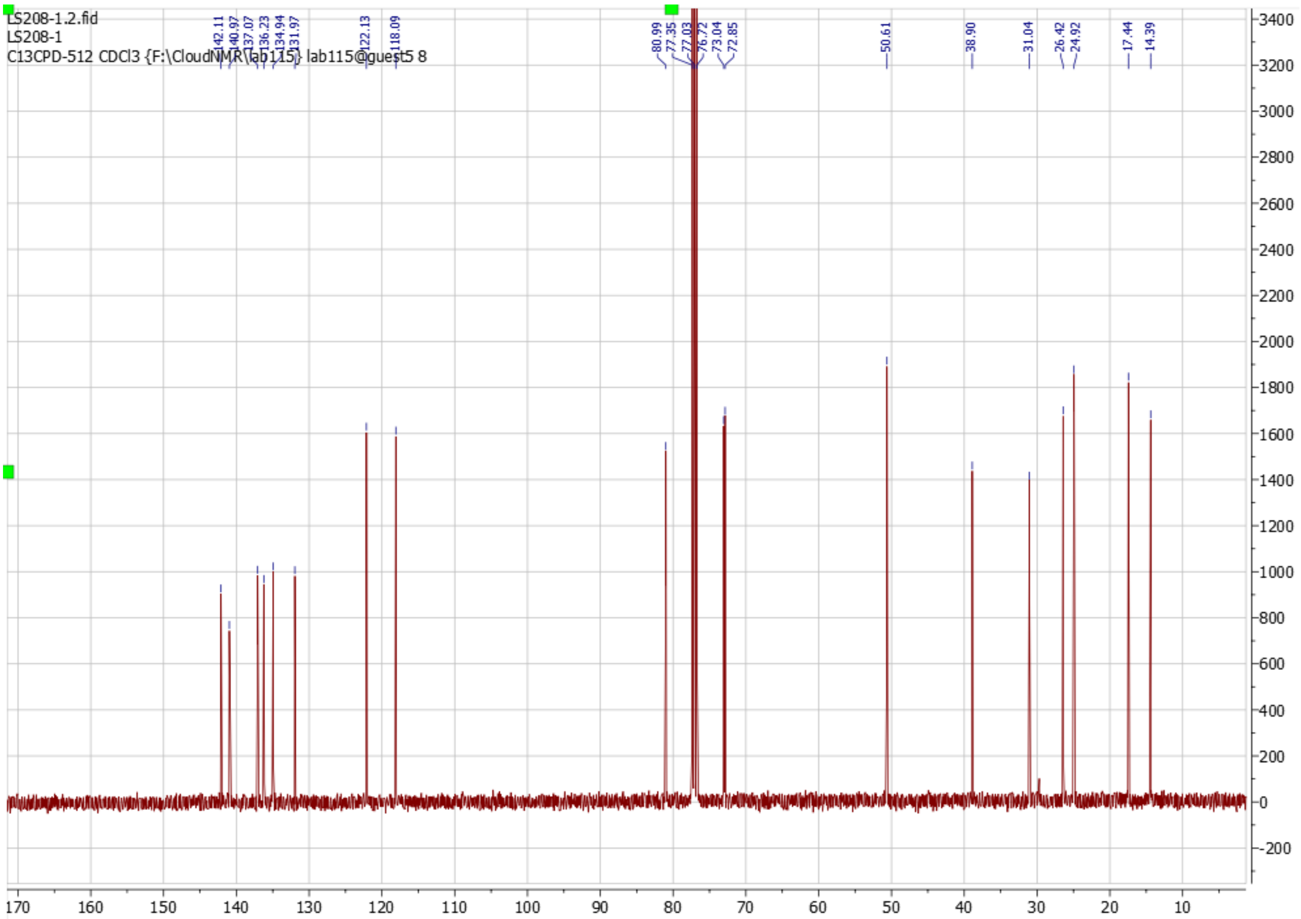

6

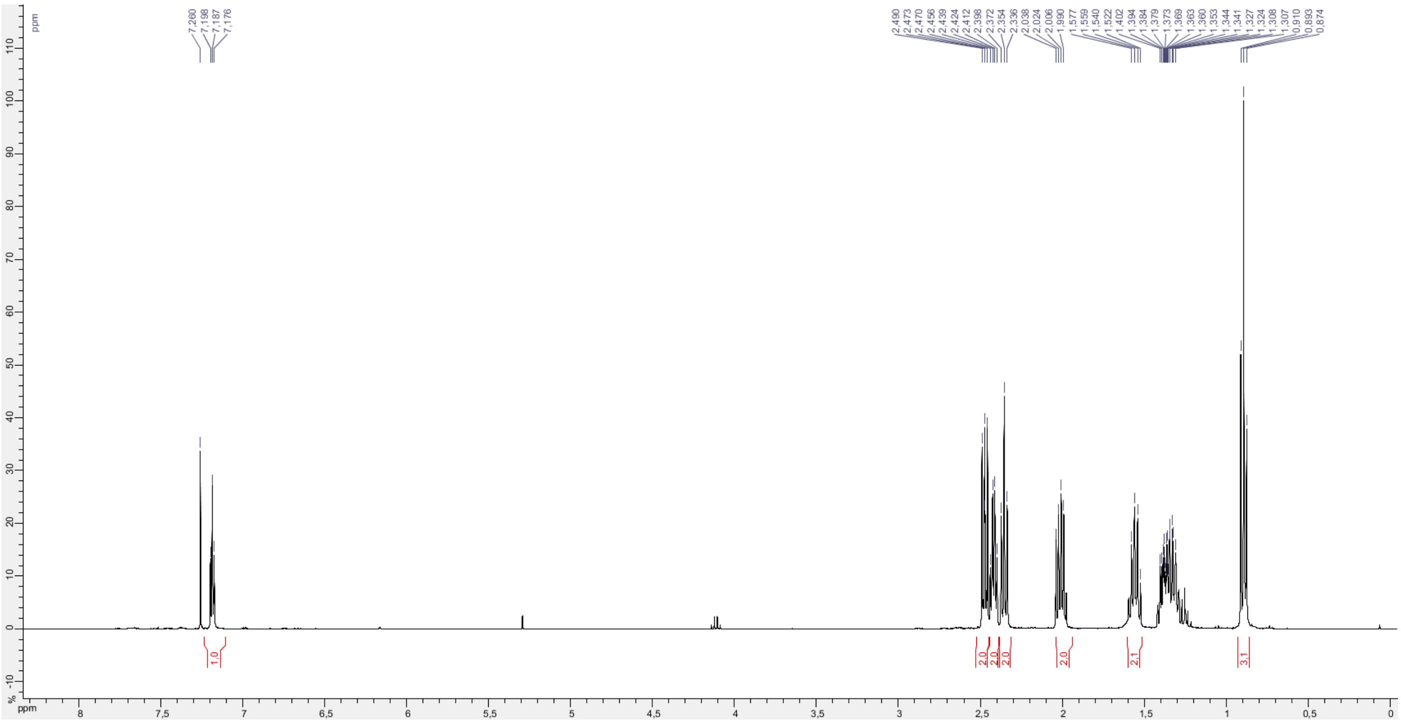
7,260
7,198
7,187
7,176
2,490
2,473
2,470
2,456
2,439
2,424
2,412
2,398
2,372
2,354
2,336
2,038
2,024
2,006
1,990
1,577
1,559
1,540
1,522
1,402
1,394
1,384
1,379
1,373
1,369
1,363
1,360
1,353
1,344
1,341
1,327
1,324
1,308
1,307
0,910
0,893
0,874
1,0
2,0
2,0
2,0
2,0
2,1
3,1
ppm

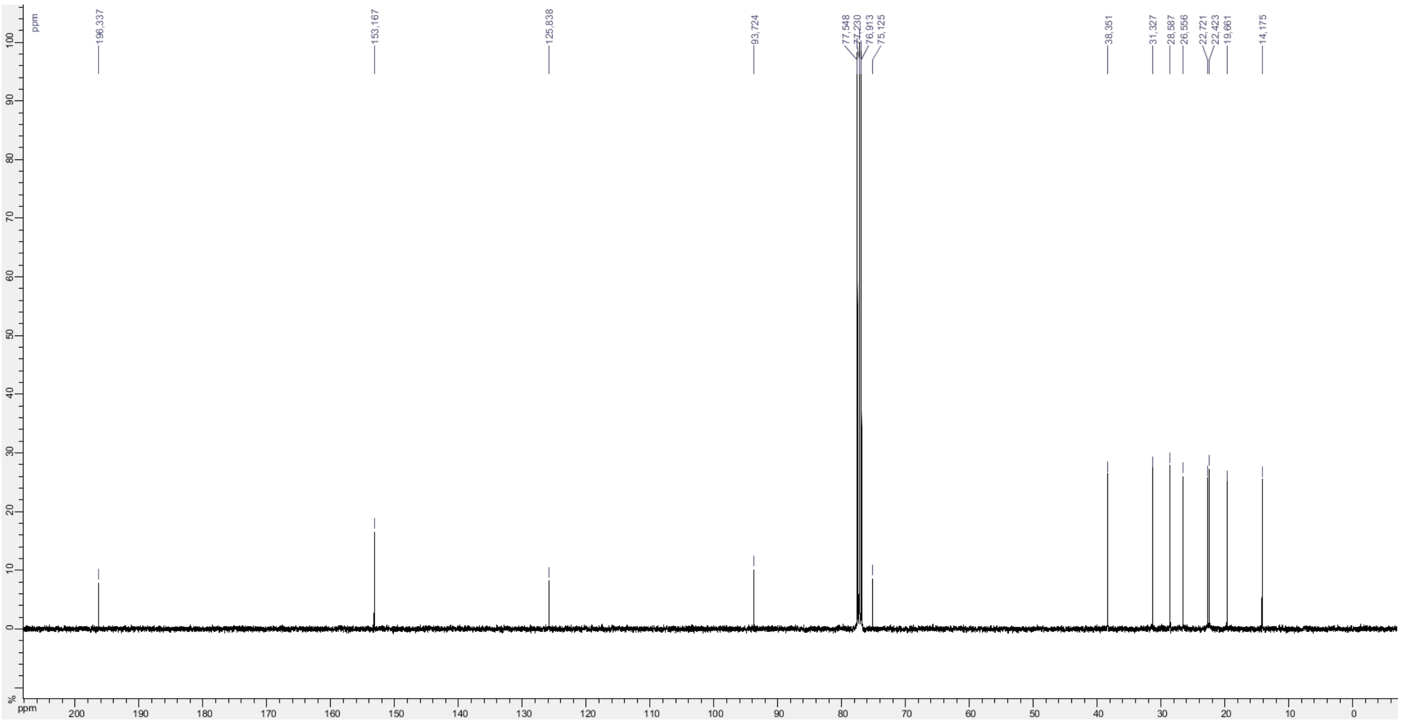
196,337
153,167
125,838
93,724
77,548
77,230
76,913
75,125
38,351
31,327
28,587
26,556
22,721
22,423
19,661
14,175
ppm

8

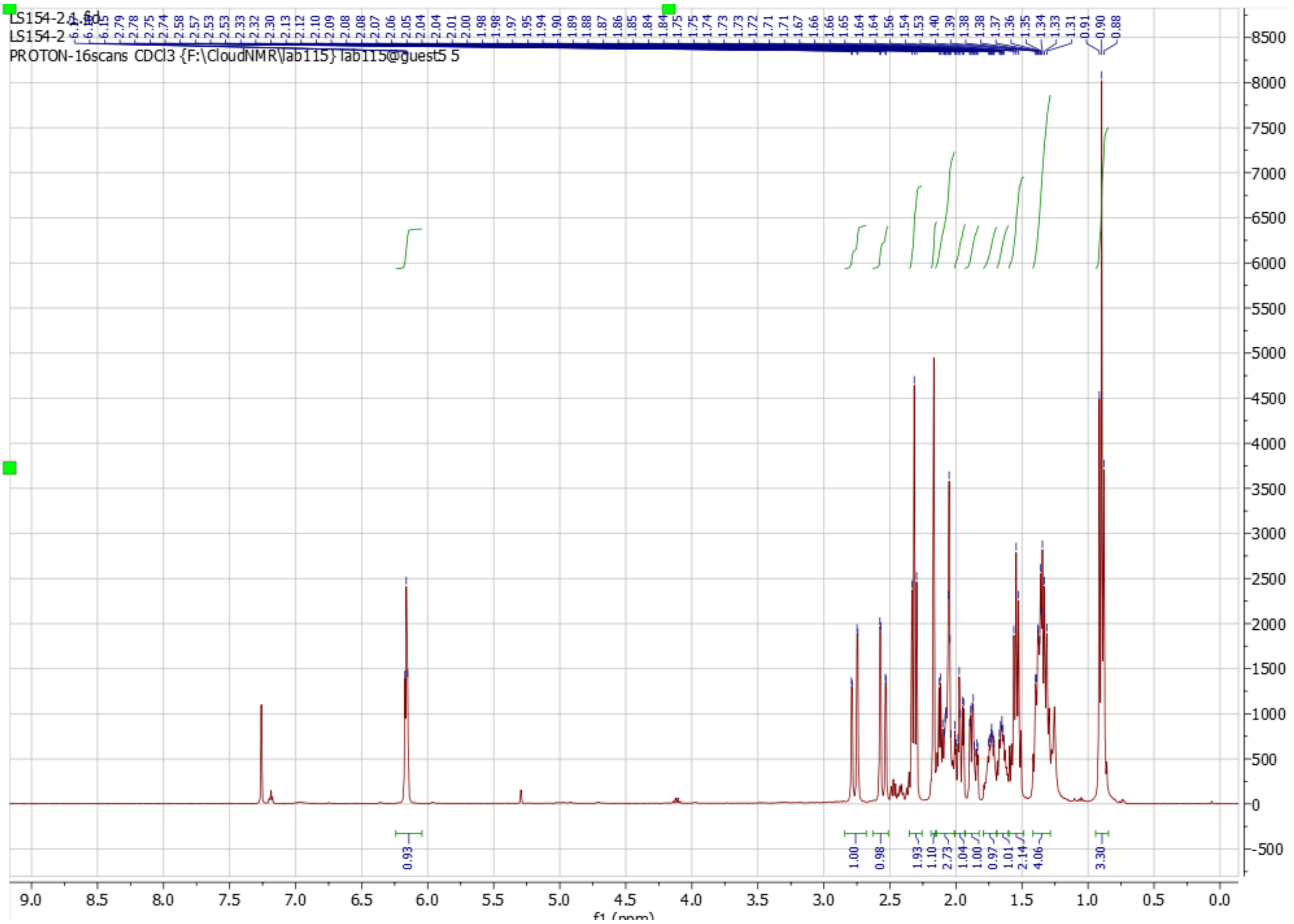
LS154-2.1.fid
LS154-2
PROTON-16scans CDCl3 {F:\CloudNMR\lab115} lab115@guest5 5
6.15
2.79 2.78 2.75 2.74 2.58 2.57 2.53 2.53 2.33 2.32 2.30 2.13 2.12 2.10 2.09 2.08 2.08 2.07 2.06 2.05 2.04 2.04 2.01 2.00 1.98 1.98 1.97 1.95 1.94 1.90 1.89 1.88 1.87 1.86 1.85 1.84 1.84 1.75 1.75 1.74 1.73 1.73 1.72 1.71 1.71 1.67 1.66 1.65 1.64 1.64 1.56 1.54 1.53 1.40 1.39 1.38 1.38 1.37 1.36 1.35 1.34 1.33 1.31 0.91 0.90 0.88
0.93
1.00
0.98
1.93
1.10
2.73
1.04
1.00
0.97
1.01
2.14
4.06
3.30
f1 (ppm)

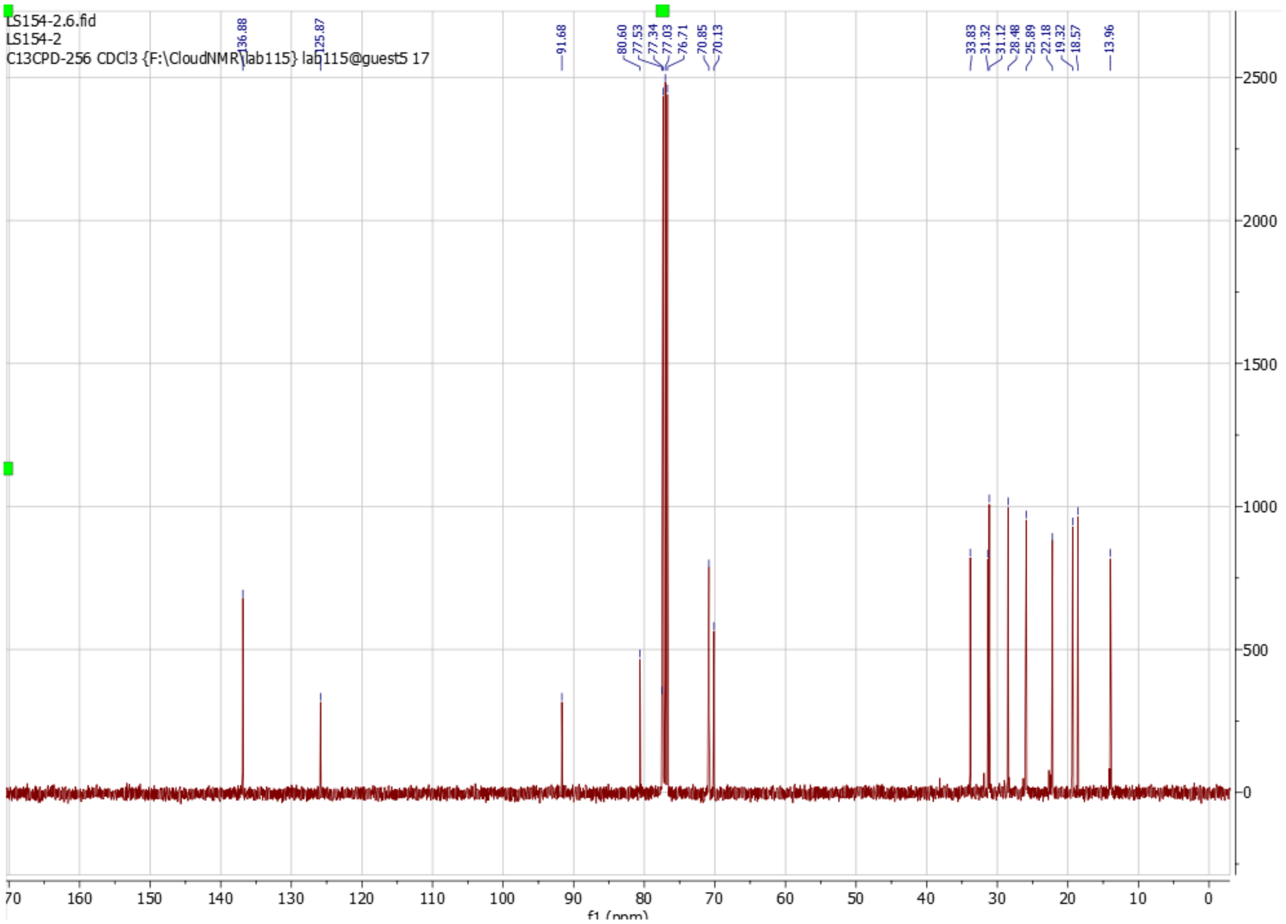
LS154-2.6.fid
LS154-2
C13CPD-256 CDCl3 {F:\CloudNMR\lab115} lab115@guest5 17
136.88
125.87
91.68
80.60 77.53 77.34 77.03 76.71
70.85 70.13
33.83 31.32 31.12 28.48 25.89 22.18 19.32 18.57
13.96
f1 (ppm)

9

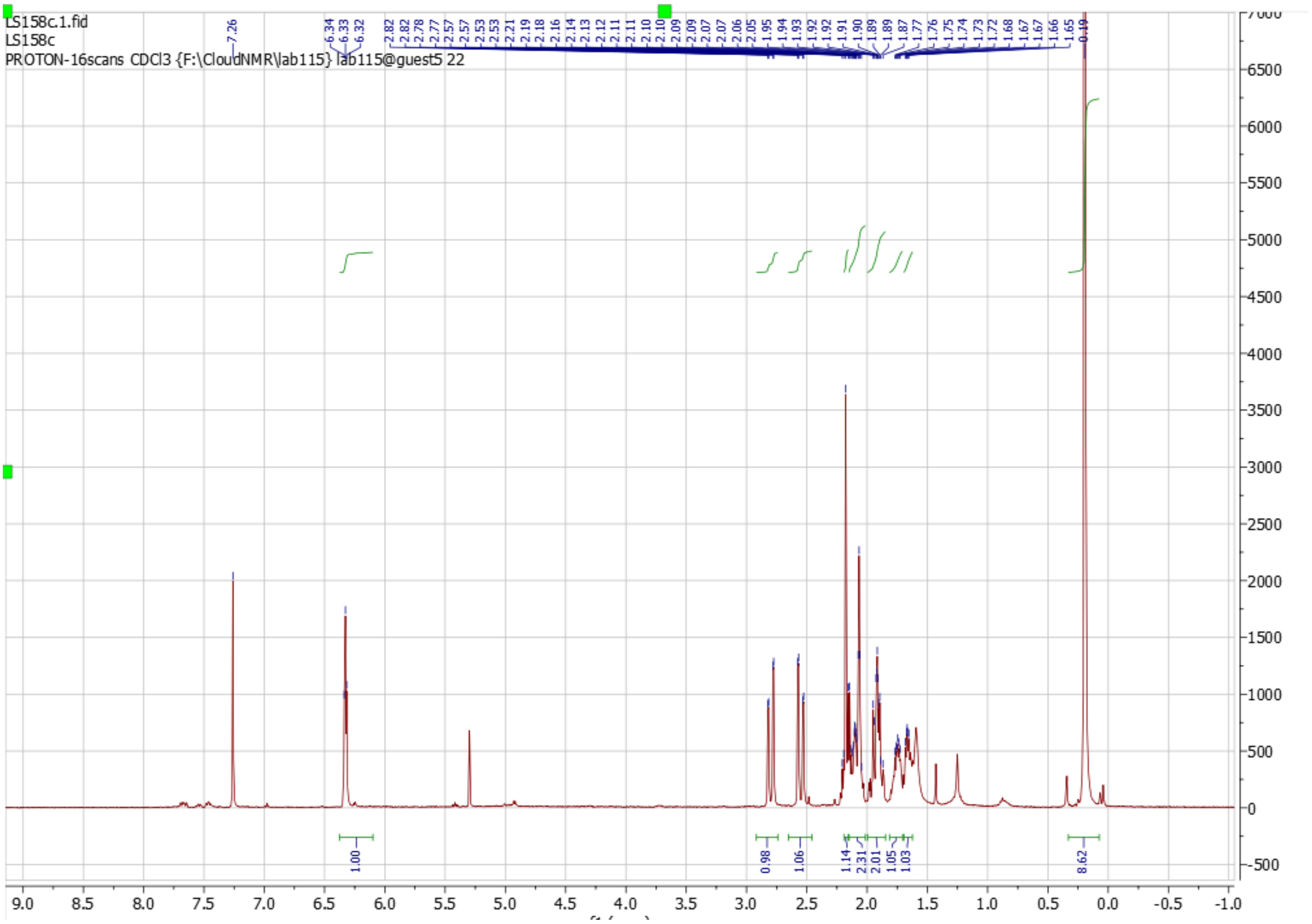
LS158c.1.fid
LS158c
PROTON-16scans CDCl3 {F:\CloudNMR\lab115} lab115@guest5 22
7.26
6.34 6.33 6.32
2.82 2.82 2.78 2.77 2.57 2.57 2.53 2.53 2.21 2.19 2.18 2.16 2.14 2.13 2.12 2.11 2.11 2.10 2.10 2.09 2.09 2.07 2.07 2.06 2.05 1.95 1.94 1.93 1.92 1.92 1.91 1.90 1.89 1.89 1.87 1.77 1.76 1.75 1.74 1.73 1.72 1.68 1.67 1.67 1.66 1.65
0.19
1.00
0.98
1.06
1.14
2.31
2.01
1.05
1.03
8.62

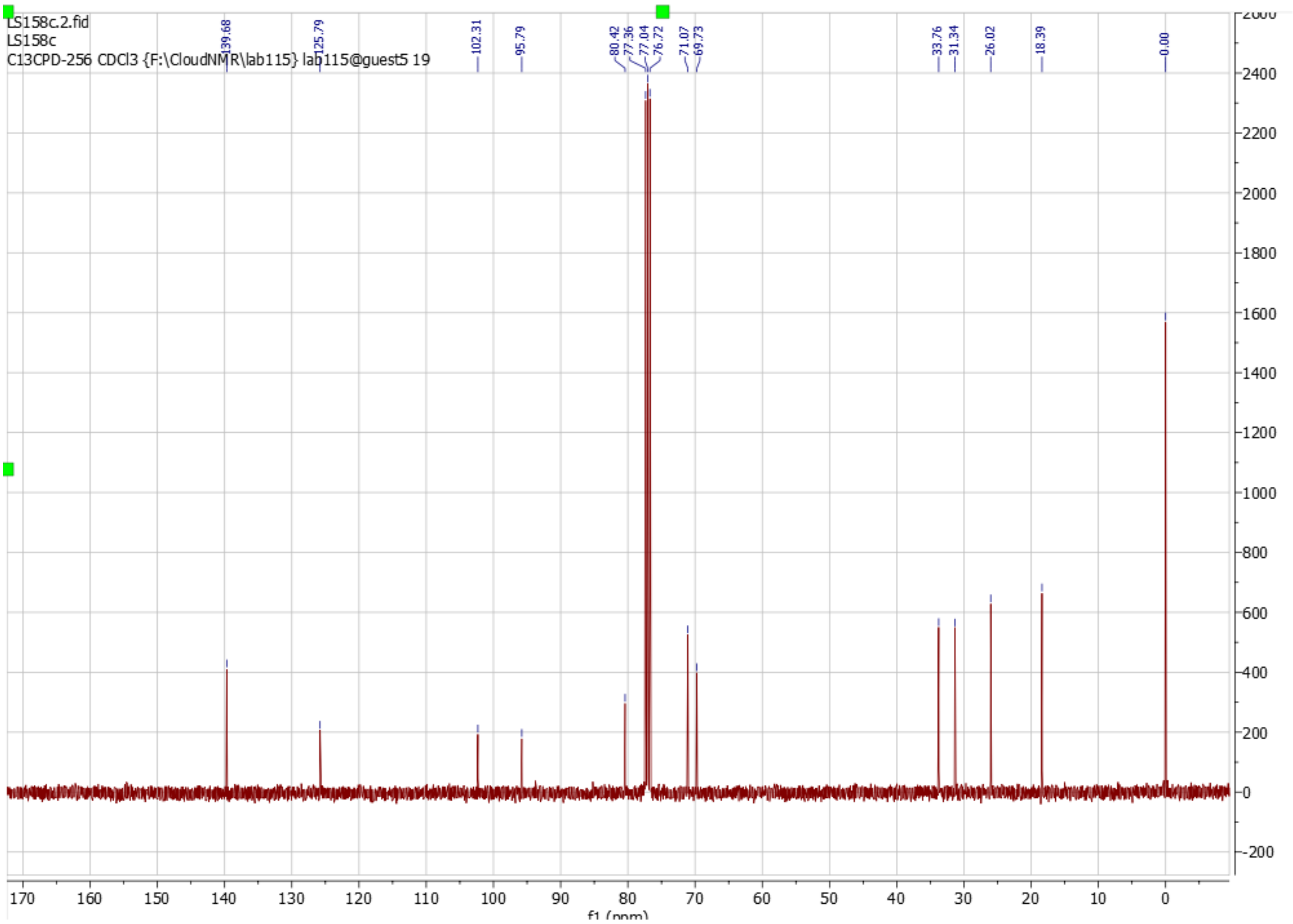
LS158c.2.fid
LS158c
C13CPD-256 CDCl3 {F:\CloudNMR\lab115} lab115@guest5 19
139.68
125.79
102.31
95.79
80.42 77.36 77.04 76.72
71.07 69.73
33.76
31.34
26.02
18.39
0.00
f1 (ppm)

10

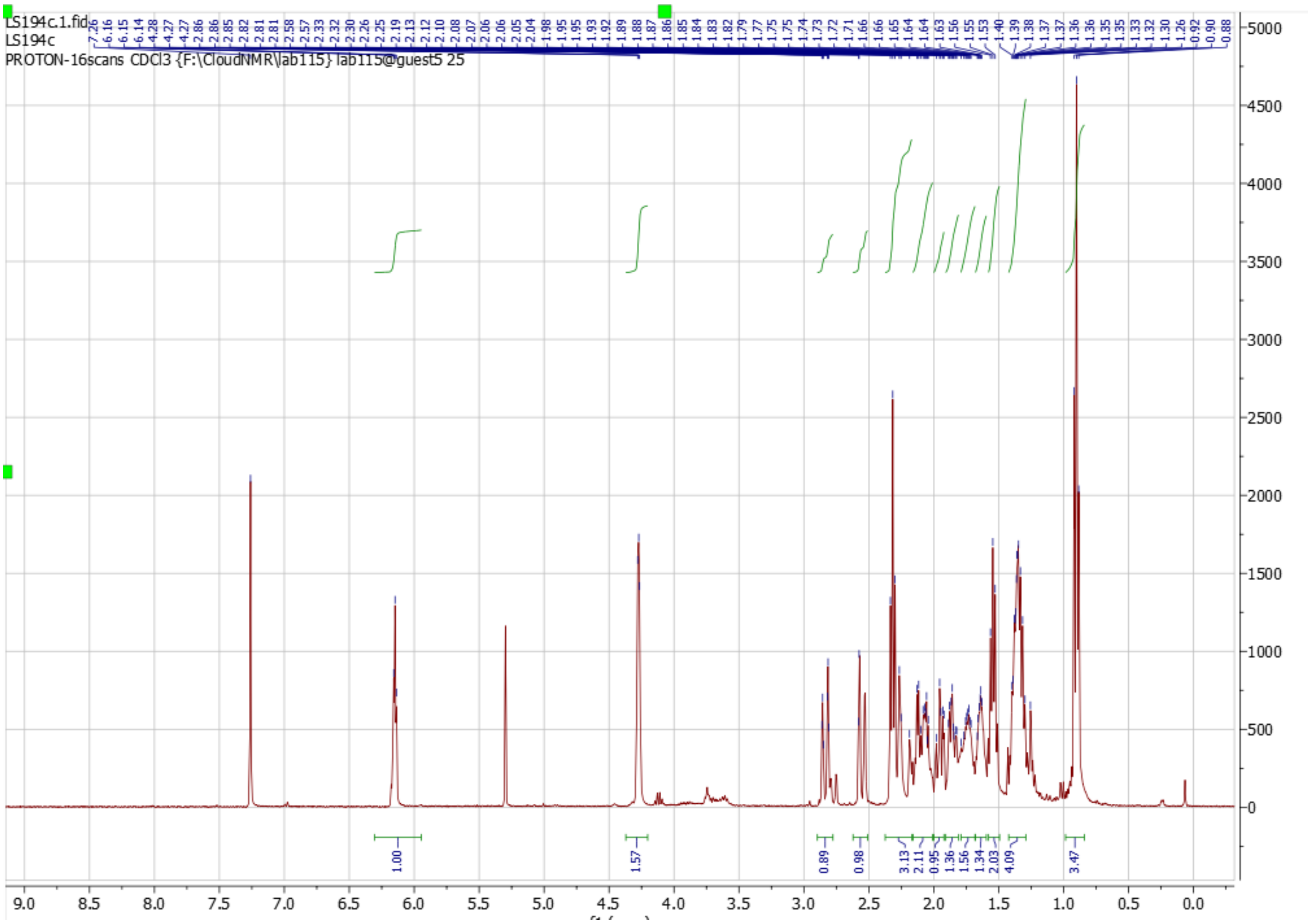

LS194c.1.fid
LS194c
PROTON-16scans CDCl3 {F:\CloudNMR\lab115} lab115@guest5 25
1.00
1.57
0.89
0.98
3.13
2.11
0.95
1.36
1.56
1.34
2.03
4.09
3.47

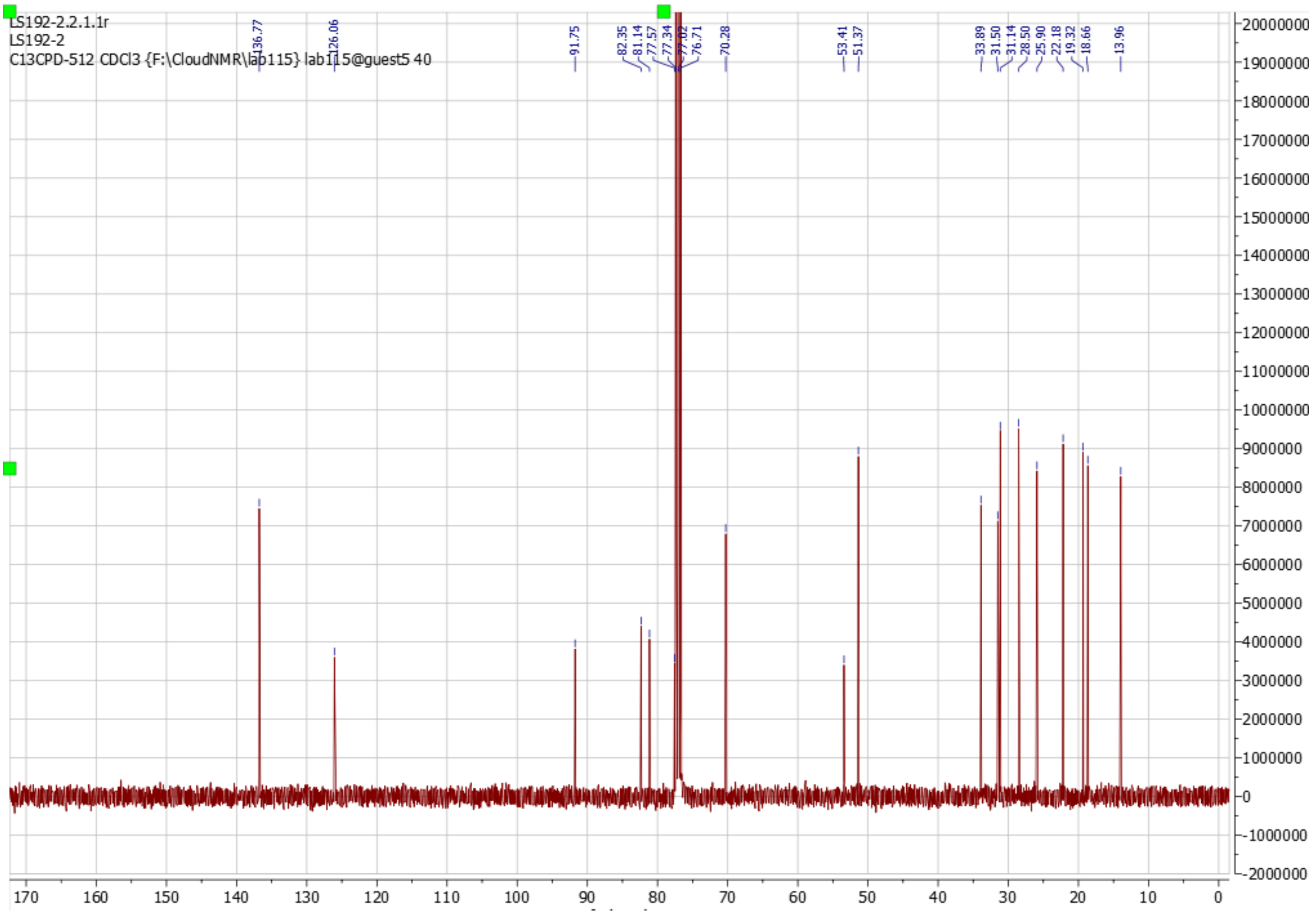

LS192-2.2.1.1r
LS192-2
C13CPD-512 CDCl3 {F:\CloudNMR\lab115} lab115@guest5 40
136.77
126.06
91.75
82.35
81.14
77.57
77.34
77.02
76.71
70.28
53.41
51.37
33.89
31.50
31.14
28.50
25.90
22.18
19.32
18.66
13.96

11

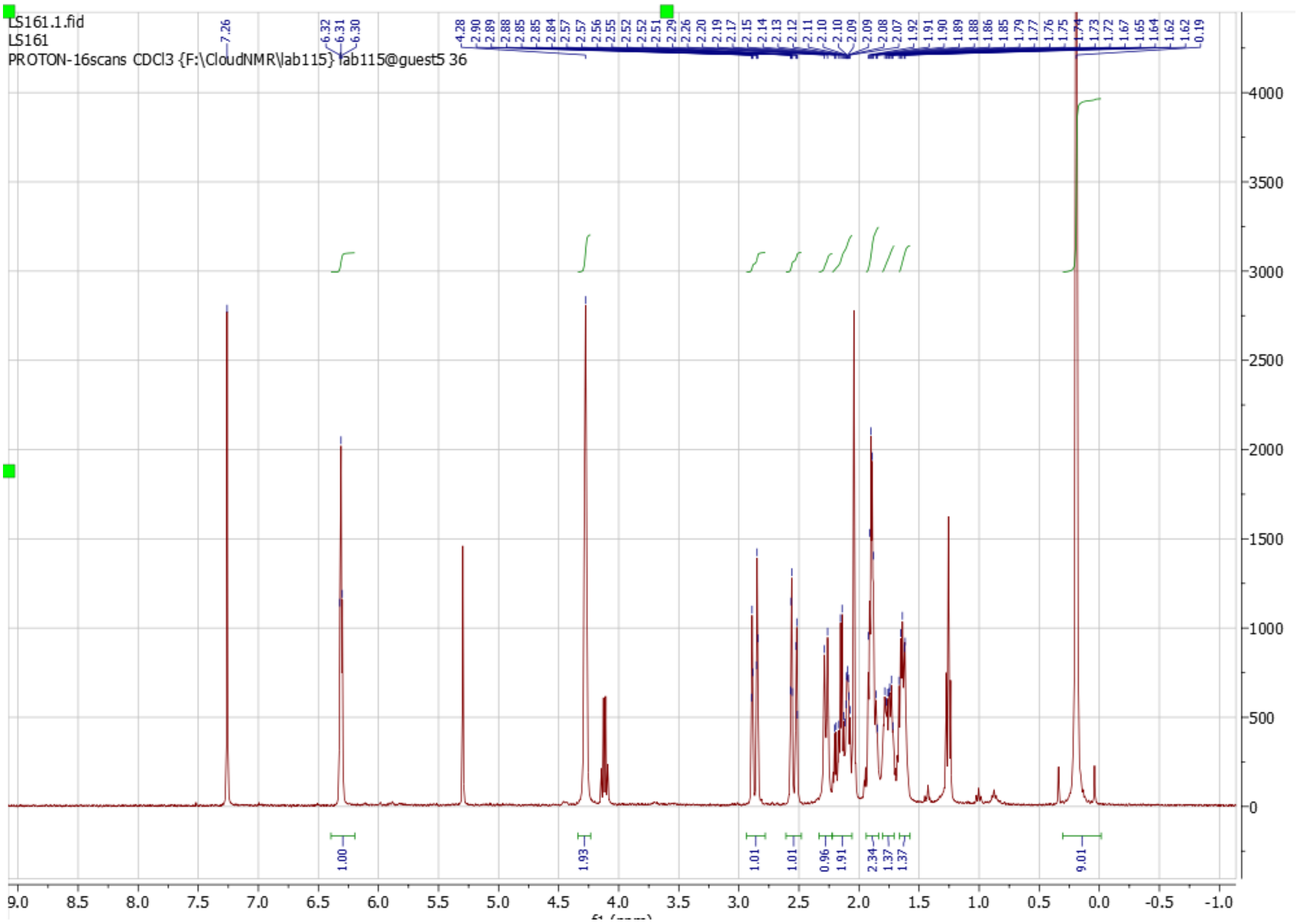
LS161.1.fid
LS161
PROTON-16scans CDCl3 {F:\CloudNMR\lab115} lab115@guest5 36

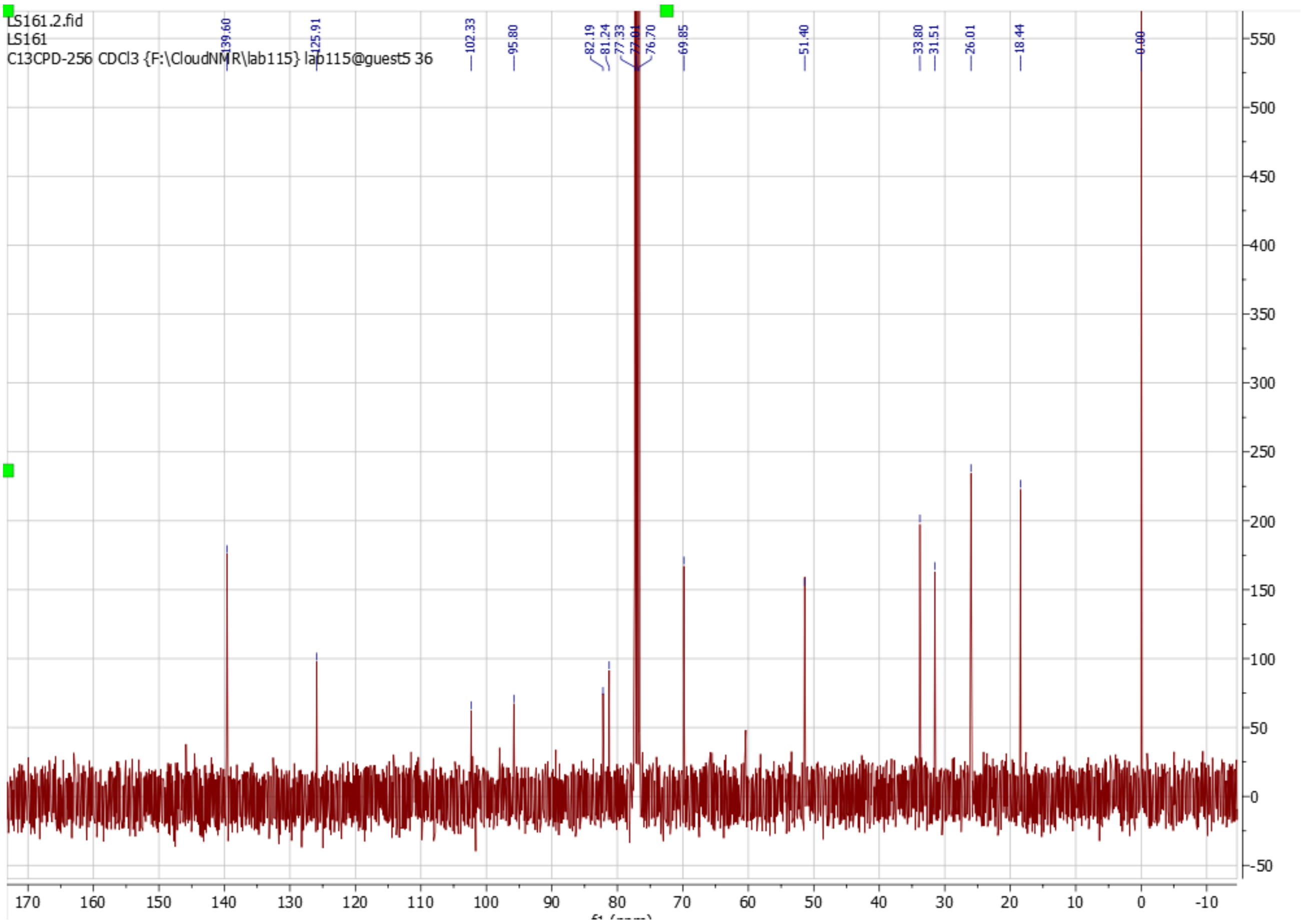
LS161.2.fid
LS161
C13CPD-256 CDCl3 {F:\CloudNMR\lab115} lab115@guest5 36

12

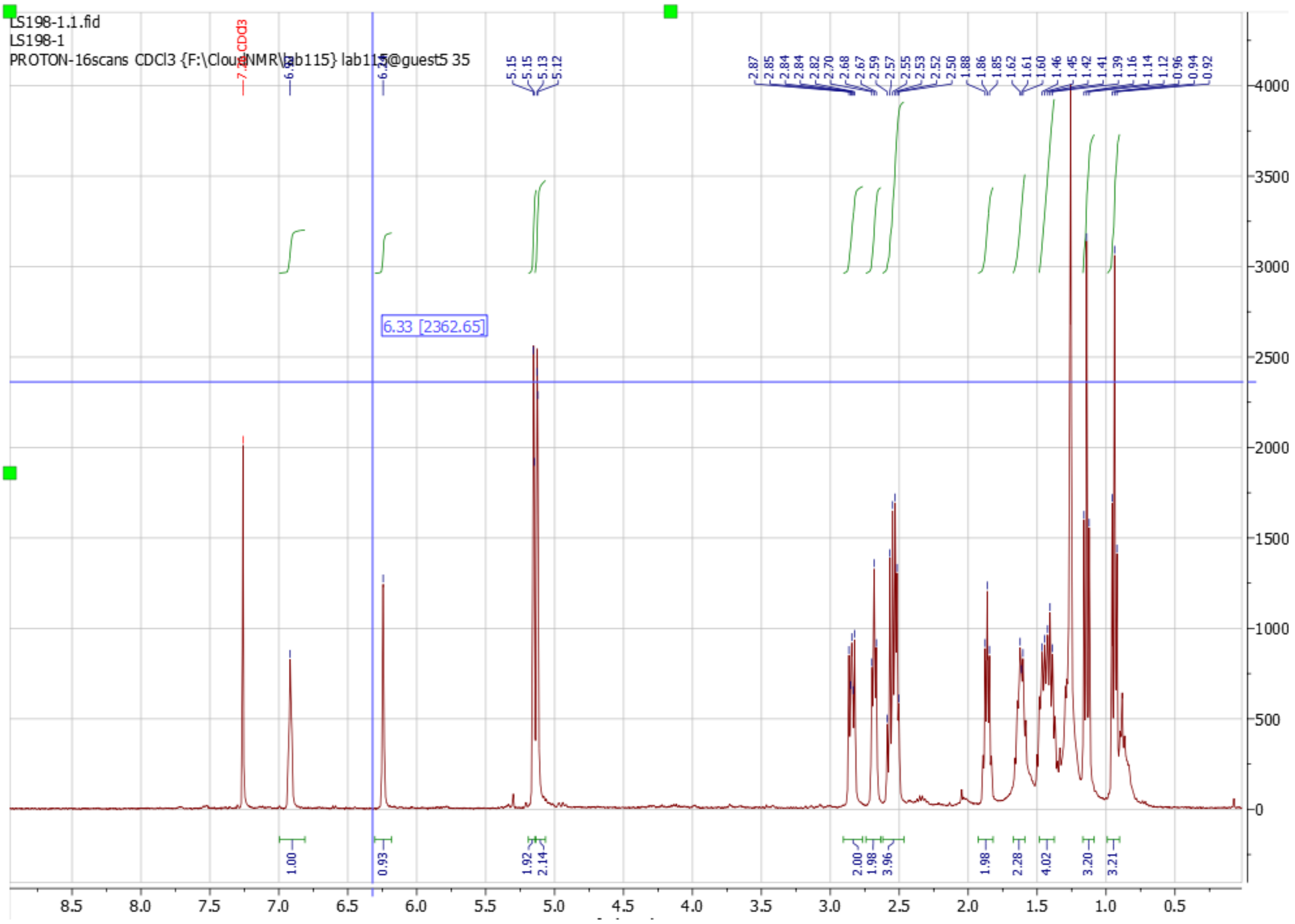

LS198-1.1.fid
LS198-1
PROTON-16scans CDCl3 {F:\CloudNMR\lab115} lab115@guest5 35
6.33 [2362.65]

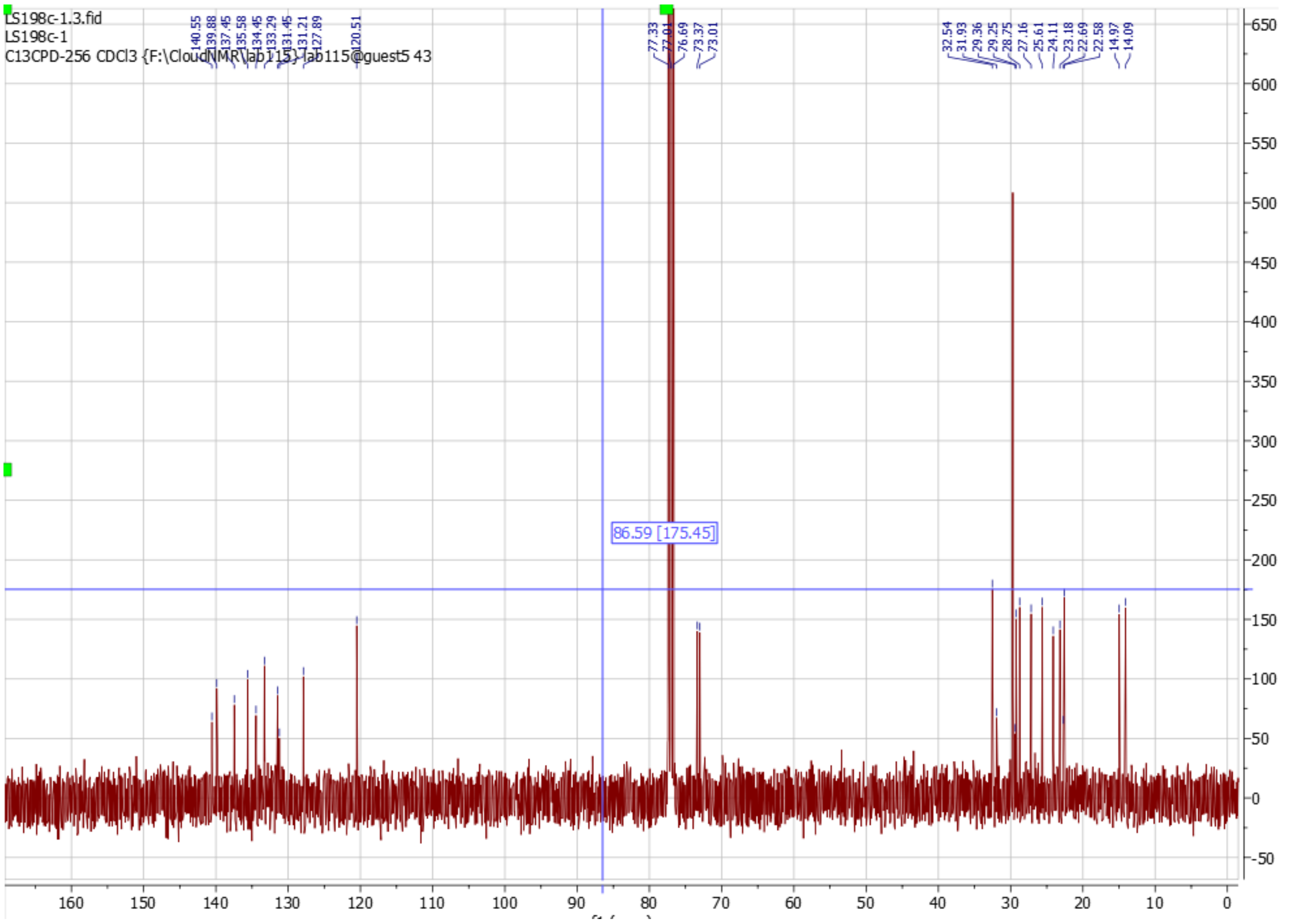

LS198c-1.3.fid
LS198c-1
C13CPD-256 CDCl3 {F:\CloudNMR\lab115} lab115@guest5 43
86.59 [175.45]

References

1. Krafft, M.E.; Cran, J.W. A convenient protocol for the iodination of unsaturated carbonyl compounds with I2 in an aqueous medium. *Synlett* **2005**, 1263–1266.

2. Lechuga-Eduardo, H.; Romero-Ortega, M.; Olivo, H.F. Synthesis of Functionalized Ring C of Escobarines. *European J. Org. Chem.* **2016**, *2016*, 51–54.
